# A Systematic Literature Review of Reproductive Toxicological Studies on Phthalates

**DOI:** 10.3390/ijms26188761

**Published:** 2025-09-09

**Authors:** Muhammad Moghazy, Marianthi Papathanasiou, Haralampos Tzoupis, Konstantinos D. Papavasileiou, Chen Xing, Volker M. Lauschke, Antreas Afantitis, Georgia Melagraki

**Affiliations:** 1 Department of Cheminformatics, NovaMechanics MIKE, 185 45 Piraeus, Greece; muhammadmoghazy@yahoo.com (M.M.); papavasileiou@novamechanics.com (K.D.P.); afantitis@novamechanics.com (A.A.); 2 Medical School, National and Kapodistrian University of Athens, 115 27 Athens, Greece; 3 Division of Physical Sciences & Applications, Hellenic Military Academy, 16673 Vari, Greece; papathanasioum98@gmail.com; 4 Department of Cheminformatics, NovaMechanics Ltd., Nicosia 1070, Cyprus; tzoupis@novamechanics.com; 5 Dr. Margarete Fischer-Bosch Institute of Clinical Pharmacology, Auerbachstraße 112, 70376 Stuttgart, Germany; chen.xing@ikp-stuttgart.de (C.X.); volker.lauschke@ki.se (V.M.L.); 6 Geschwister-Scholl-Platz, University of Tübingen, 72074 Tübingen, Germany; 7 Department of Physiology and Pharmacology and Center for Molecular Medicine, Karolinska Institute and University Hospital, 171 76 Stockholm, Sweden; 8 Department of Pharmacy, The Second Xiangya Hospital, Central South University, 139 Renmin, Changsha 410011, China; 9 Entelos Institute, Nicosia 2102, Cyprus

**Keywords:** reproductive health, phthalates, reproductive toxicology, phthalate toxicity, di(2-ethylhexyl) phthalate (DEHP), dibutyl phthalate (DBP), phthalate exposure

## Abstract

Phthalates are widely used plasticizers recognized as endocrine-disrupting chemicals (EDCs) with well-documented adverse effects on reproductive health. These compounds act either directly or through their metabolites and can influence various biochemical pathways. Key phthalates that have been associated with potential toxic outcomes include di(2-ethylhexyl) phthalate (DEHP), dibutyl phthalate (DBP), butyl benzyl phthalate (BBP), diisononyl phthalate (DiNP), and diisodecyl phthalate (DiDP). The presence of these compounds in everyday consumer products has been associated with various adverse effects on human reproductive health, including hormonal disruption, issues in gonadal function, and other hormone related problems. This systematic review provides an overview and critical synthesis of the most recent research regarding phthalate reproductive toxicity. The scope is to summarize and aggregate correlations between phthalate exposure and reproductive health outcomes and highlight factors, such as age, sex, and extent of exposure, that have the most significant impacts on clinical outcomes. The reported studies focus on the gender-specific outcomes of various phthalates, while the epidemiological data reveal the importance of exposure duration and age. The reported results highlight the need for strict regulations regarding phthalate usage and the importance of developing safer alternatives.

## 1. Introduction

Endocrine-disrupting chemicals (EDCs) are substances that bind to protein targets inside the cells and induce adverse health effects in the human organism [1,2,3]. EDCs exert their action by targeting hormone synthesis and thus causing disruptions in the endocrine system [4]. These chemicals have been linked to dysfunctions in the reproductive system and developmental disorders in humans [5]. The compounds have been shown to interact with various nuclear receptors (e.g., estrogen and androgen receptors, pregnane X receptor) [6] and thus show variability in their action. One such group of EDCs contains phthalates, the diesters of orthophthalic acid (1,2-benzenedicarboxylic acid). These chemicals are a broad class of synthetic compounds, which have been widely used for decades as plasticizers to improve the elasticity and workability of plastics across several industries [7]. Due to their non-covalent bonding with plastic materials, phthalates can leach into the environment, through air or dust, leading to widespread human exposure through ingestion, inhalation, or dermal contact [8,9]. Phthalates have been the focus of environmental and public health concerns due to their well-documented endocrine-disrupting activity [10,11]. Safety concerns regarding the adverse reproductive effects related to these chemicals have stirred a multitude of in vitro, in vivo, and epidemiological studies over the last few years.

Phthalates are inexpensive to produce, readily incorporated into polymers, and can improve product performance [11,12,13]. Their primary use is to improve the flexibility and durability of polyvinyl chloride (PVC) products by acting as plasticizers [14]. According to the U.S. Environmental Protection Agency (EPA) and the European Chemicals Agency (ECHA), these compounds are commonly found in various consumer products, including toys, textiles, food packaging, personal care items, and medical devices [15,16]. Phthalates, such as dibutyl phthalate (DBP) and diethyl phthalate (DEP), are used extensively as excipients in human pharmaceutical products. They are utilized as film-forming agents in coating drugs to facilitate controlled release [17]. The European Medicines Agency has issued exhaustive scientific guidelines regarding the use of phthalates in drug formulations and associated safety considerations [18]. Phthalates are not chemically bound to polymer matrices [19,20]. Their inherent physicochemical properties, such as high diffusivity, facilitate leaching and subsequent migration into the environment, resulting in widespread exposure [21].

The compounds can be categorized into two primary groups according to their molecular weight: high molecular weight (HMW) phthalates, utilized to enhance the elasticity of polyvinyl chloride, and low molecular weight (LMW) phthalates, applied as adhesives or solvents in the production of cosmetic products [22,23,24]. Their metabolism consists of two biotransformation phases, which are hydrolysis and conjugation [25]. During hydrolysis, diester phthalates are converted into monoesters by carboxylesterases (e.g., CES1, CES2) and lipase enzymes (e.g., pancreatic lipase). This process could lead to metabolites with increased bioactivity [25,26]. Following this, the second phase of conjugation usually involves the UDP-glucuronosyl-transferase enzyme, which creates hydrophilic conjugates that can be readily eliminated through urine [25].

Exposure to phthalates begins in utero and continues throughout a person’s lifetime via dietary sources, dermal absorption, and inhalation [27]. The occurrence of phthalates and their metabolites in human biological samples, including urine, serum, breast milk, and semen, has been widely reported [28], which raises concerns about possible health implications. Researchers have identified several of these compounds as reproductive and developmental toxicants in animals [29], and their role as endocrine disruptors in humans is extensively recorded [30,31,32]. Additionally, human epidemiological studies relate exposure to phthalates to negative reproductive outcomes in both males and females [14]. Determining the overall effect of phthalates on reproductive health requires a detailed understanding of the exposure pathways.

Phthalate exposure has been frequently associated with reproductive health issues in both males and females [33,34]. According to previous reports in the literature, compounds such as di-(2-ethylhexyl) phthalate (DEHP) can decrease sperm motility, impede sperm maturation, and lower blood testosterone levels [35,36]. Additionally, exposure to phthalates may potentially increase the risk of infertility by altering the size and function of male reproductive organs, such as the prostate and testes [37]. In females, phthalate exposure is primarily associated with hormonal imbalances that may contribute to ovarian dysfunction. Some of their main effects include decreased estradiol levels, ovarian morphological changes, reduced ovarian reserve, and anovulation [37]. Moreover, they have been shown to disrupt follicle development, increase oxidative stress, and accelerate follicular degeneration, prompting ongoing investigations into their long-term effects on female fertility [38]. However, further research is required to fully elucidate the extent of phthalate-related reproductive toxicity.

Given the serious reproductive health risks associated with phthalates, regulatory agencies have initiated actions to address these rising safety concerns. In the United States, the Consumer Product Safety Commission (CPSC) has banned the use of phthalates in toys and childcare items since 2018 [39]. Similarly, the EU has implemented restrictions under the REACH regulations, classifying certain phthalates, such as DEHP, DBP, di-iso-butyl phthalate (DiBP), and benzyl butyl phthalate (BBP), as substances of very high concern (SVHC). Since November 2020, several phthalates, including DEHP, DBP, DiBP, BBP, di-iso-pentyl phthalate (DIPP), di(2-methoxyethyl) phthalate (DMEP), di-n-pentyl phthalate (DPP), di-n-hexyl phthalate (DnHP), and 1,2-benzenedicarboxylic acid, have been restricted in the EU due to their classification as toxic. These restrictions apply to consumer clothing, accessories, and other textiles that come into contact with the skin [16]. Furthermore, in response to a 2018 request from the Flexible Vinyl Alliance, the FDA has rescinded the permission for the use of 23 phthalates that are no longer utilized, thereby reducing the list of approved phthalates used in food packaging to nine [40].

Despite considerable efforts to mitigate the risks associated with phthalate exposure, understanding their long-term effects and evaluating the overall effectiveness of regulatory interventions remains challenging. Prior literature reviews have established a solid understanding of the reproductive toxicities associated with phthalates. For example, one review examined in vitro, in vivo, and epidemiological data to demonstrate how phthalates (i) disrupt the hypothalamic–pituitary–gonadal (HPG) axis, (ii) interfere with steroid production, and (iii) alter receptor-mediated cellular pathways [37]. Other reviews categorized phthalates according to their molecular weights and showed how each group is associated with specific adverse reproductive effects in both males and females, while elucidating key mechanisms such as oxidative stress and anti-androgenic effects [33]. Nonetheless, significant gaps in the current literature remain, especially the lack of an updated integrative review that incorporates recent findings as well as implementing a systematic approach to assess heterogeneous data across different study designs, exposure levels, and endpoints.

The present systematic review addresses these gaps by integrating evidence gathered from January 2020 to June 2024 through a PRISMA (Preferred Reporting Items for Systematic reviews and Meta-Analyses)-guided, methodologically robust evaluation, thereby providing a more comprehensive analysis of phthalates’ reproductive toxicity while ensuring transparency and reproducibility. We provide a summary of the available evidence on phthalate exposure, metabolism, and reproductive toxicity from in vivo, in vitro, and epidemiological studies with a particular emphasis on the reproductive toxicities associated with phthalates (e.g., impacts on fertility, hormonal disruption, and overall reproductive health). By systematically reviewing the recently published literature, this work aims to update existing knowledge on phthalate-related reproductive toxicity drawing on reported toxicity profiles of individual compounds. The scope is to address key questions such as the following: “*What reproductive toxicity outcomes are linked to phthalate exposure?*” and “*Which phthalates present the strongest evidence of inducing adverse reproductive impacts?*”. By combining data on the chemical structure of phthalates, exposure routes, mechanistic toxicology studies, and the regulatory actions taken to restrict their use, the review presents the associated mechanisms by which phthalates influence reproductive health and determines whether current risk reduction measures are sufficient. Moreover, it aims to present some of the most important findings that may inform future research agendas. Ultimately, the results of this study could guide the formulation of a more robust public health policy with revisions to existing regulatory frameworks. Additionally, this study could aid in the development of less toxic chemical options, thereby reducing human exposure to phthalates and protecting reproductive health. This review is strictly descriptive and does not quantify dose–response relationships across studies. We acknowledge that our focus on studies from 2020 to 2024 might limit the ability to perform a meta-analysis and may introduce selection bias.

## 2. Methodology

### 2.1. Eligibility Criteria

This review includes studies published in English between January 2020 and June 2024 that investigate the reproductive toxicity of phthalates, either as single compounds or in mixtures (Table S1). The systematic review was registered on the OSF database [https://osf.io/ (accessed on 1 August 2025)] for systematic reviews with the DOI identifier 10.17605/OSF.IO/R2SW4. Table 1 lists typical phthalates with their primary and secondary metabolites. Table 2 shows the most common phthalates reviewed in the literature, with DEHP, DBP, and BBP receiving the most attention due to their widespread use and regulatory limitations. The structural diversity of phthalates, reflected in variations in molecular weight and composition, may influence their environmental persistence and toxicity.

Both human and animal studies were included, with focus on in vivo models relevant to human reproductive health, including rats and mice. In vitro studies using human cell lines and samples were also included to assess the cell specificity of the toxicity impact and thus improve the accuracy of extrapolating human health effects. The inclusion criteria required that studies report direct parental reproductive toxicity, involving at least one of the following outcomes: fertility parameters (e.g., sperm quality, follicular count), reproductive health (e.g., hormonal levels, testicular or ovarian function), or reproductive toxicity. Both experimental and observational study designs (e.g., cohort, case–control, cross-sectional) qualified for inclusion.

The following exclusion criteria were applied:

- **Language**: Studies not published in English.
- **Studies with non-conserved reproductive mechanisms**:
  o In vitro studies using animal cell lines.
  o In vivo studies employing animal models where experimental endpoints lack translational validity for human reproductive health (e.g., zebrafish (*Danio rerio*) and nematodes (*C. elegans*)).
- **Co-exposure**: Studies investigating phthalate exposure in combination with other chemicals.
- **Irrelevant outcomes:** Studies that did not directly report on fertility, reproductive health, or reproductive toxicity outcomes, such as research on gestational diabetes, fetal development, or pregnancy outcomes.
- **Non-original research:** Case reports, case series, conference papers, editorials, opinion pieces, letters to the editor, reviews, and meta-analyses.

### 2.2. Search Strategy

The aim of this systematic review was to gain insights into the reproductive toxicities of phthalates. PubMed and Scopus were selected as the primary databases. Gray literature (e.g., theses, conference proceedings) was excluded to focus exclusively on fully peer-reviewed studies. The search terms that were used to search abstracts, titles, and keywords of papers were the following: (“phthalates” OR “phthalate esters” OR “DEHP” OR “DBP” OR “BBP” OR “DiNP” OR “DiDP”) AND (“fertility” OR “reproductive health” OR “infertility” OR “reproductive toxicity” OR “sperm count” OR “ovarian function” OR “hormonal disruption” OR “pregnancy outcomes”). This combination of search terms was used to retrieve a wide range of relevant studies that could be evaluated based on the specified inclusion criteria. While the term “pregnancy outcomes” was included in the search strategy to ensure comprehensive retrieval of potentially relevant studies, such research whose sole endpoints were pregnancy outcomes or fetal development was later excluded during screening based on predefined exclusion criteria (see Section 2.1). However, any study that assessed at least one parental reproductive endpoint was retained even if it also reported pregnancy data. The initial search spanning articles from January 2020 to June 2024 yielded 477 PubMed and 649 Scopus results, for a total of 1126 publications.

### 2.3. Study Selection Process

The study selection process was systematically conducted. Zotero—Corporation for Digital Scholarship: Vienna, VA, https://www.zotero.org/ (accessed 24 September 2024), was utilized to remove duplicates via the “Duplicate Items” view (v6.0.28), matching on title, author, year, and DOI (fuzzy-match threshold 0.75). Each group was manually reviewed, and we retained the record with the most complete metadata (e.g., full abstract, complete author list); all retrieved records were exported for reference. After removing 402 duplicates and one retracted record, 723 publications were assessed for eligibility. The screening process was divided into three stages: an initial title and abstract screening, followed by a refined abstract screening, and finally a full-text screening. The screening was carried out by the first author, and a second reviewer independently checked the selections to ensure consistency and accuracy. During the preliminary stage, all titles, abstracts, and keywords were screened for eligibility criteria, resulting in 420 papers left for further assessment. We then proceeded with a more targeted abstract screening aligned with our scope, resulting in the exclusion of 322 studies for the following reasons: different animal models (n = 25), mixtures and co-exposures (n = 69), no original data (n = 95), out of scope (other health risks, different chemicals, in vitro animal studies; n = 44), and pregnancy outcomes and fetal development (n = 89), which left 98 studies for full-text screening.

During the final screening process, we excluded eight studies due to the lack of access to the full-text articles. Eventually, 90 studies were included in our review for data extraction. The full selection process was documented using a PRISMA flow diagram (Figure S1), and the completed PRISMA checklist is presented in Table S2 to ensure transparency and reproducibility [41].

## 3. Results

### 3.1. Overview of Selected Studies

A total of 1126 records were retrieved from PubMed (477) and Scopus (649). A total of 90 publications met the eligibility criteria for inclusion in this systematic review as outlined in Section 2.2. The reported studies included 30 human epidemiological studies, which were further subdivided into 7 case–control, 7 cohort, and 16 cross-sectional studies, 54 studies on animal models, and 11 in vitro studies, employing human cell lines and patient samples. Some articles included a combination of studies (n = 5) such as in vivo tests on animal models and in vitro experiments on human cell lines or samples, accounting for the overlap in the total count.

All considered in vivo studies examined the effects of phthalates on reproductive health in rodent models. These studies focused mostly on endpoints such as sperm quality, ovarian function, and hormonal disruptions. Human cell lines, including human granulosa-like tumor cell line (KGN), granulosa cells (GCs), and human prostatic cell line (PNT1A), were used in the in vitro analyses to investigate the molecular mechanisms behind phthalate-induced reproductive toxicity. Moreover, human epidemiological studies provided overall insights into the impact of phthalate exposure, with a focus on reproductive outcomes such as infertility, hormonal imbalances, and semen quality parameters.

### 3.2. In Vivo Studies

The results of the in vivo studies and the toxicity outcomes related to phthalate exposure in the animal models are collected in Table 3 and Table S3. Rodents were exposed to DEHP, DEP, BBP, or other phthalates, mostly via oral routes. The toxicity outcomes reported included testicular atrophy, a decreased testicular index [42], and significant sperm quality reductions in male animals [43], and a decrease in the number of follicles, ovarian blood vessel congestion, and oocyte distortion in female animals [44]. The aforementioned toxicities have been potentially induced, among other mechanisms, through the interplay of phthalates in the androgen and estrogen signaling pathways.

In female mice, exposure to DEHP resulted in a significant decrease in ovarian weight, a reduction in serum levels of 17β-estradiol (E2), and an elevation of apoptotic markers (Caspase-9, BAX/BCL2) within ovarian cells [45,46]. Moreover, it caused an increase in the levels of the zinc transporter SLC39A5 in ovarian granulosa cells [47]. This elevation triggered the activation of the NF-κB signaling pathway, demonstrated by the phosphorylation of the p65 subunit and the breakdown of its inhibitor IκBα. Subsequently, the activated NF-κB promoted the expression of the NLRP3 gene, which resulted in inflammatory cell death (pyroptosis) of the granulosa cells, hindering follicle development and overall ovarian function [47].

On the other hand, DEHP exposure in male mice led to a reduction in testicular weight, a significant drop in sperm count, and decreased serum testosterone levels. Additionally, DEHP exposure led to the downregulation of tight junction proteins (ZO-1, connexin-43 (CX-43), N-cadherin) in Sertoli cells, which compromised the blood–testis barrier (BTB) [48,49,50]. Studies on rats also revealed that DBP exposure caused an increase in miR-506-3p expression levels, a type of microRNA, which in turn downregulated Annexin A5 (ANXA5) and suppressed the Nrf2/HO-1 signaling pathway responsible for activating antioxidant defenses in rat testes. This disruption resulted in reduced levels of antioxidant enzymes (CAT, SOD, T-AOC, GSH), while also leading to an increase in oxidative damage markers such as MDA and the accumulation of reactive oxygen species (ROS) [51].

Phthalate mixture (DEHP, DBP, BBP) exposure in male Sprague-Dawley (SD) rats further demonstrated significant reproductive toxicity, with varying outcomes based on dose and duration of exposure. In the high-dose regiment (450 mg/kg/day for 91 days), the exposure resulted in upregulation of steroidogenic enzymes such as CYP11A1, CYP17A1, and 17β-Hydroxysteroid dehydrogenase (17β-HSD), as well as decreased expression of Steroidogenic Acute Regulatory Protein (StAR), suggesting a significant disruption in steroid hormones production [52]. However, in the low-dose protocol (16 mg/kg/day for 91 days), the results showed downregulation of 17β-HSD and CYP19A1, but upregulation of StAR, CYP11A1, and CYP17A1, indicating a compensatory response to lower toxicity [53]. In addition, the high-dose study showed downregulation of PIWIL1 and PIWIL2, which are essential for spermatogenesis, whereas the low-dose study reported upregulation of PIWIL1 and downregulation of PIWIL2. This outcome indicated dose-dependent effects on germ cell maintenance and differentiation. These findings highlight the potentially complex, dose-dependent mechanisms by which phthalates disrupt male reproductive function.

**Table 3:** Overview of the main toxicological effects of phthalates on reproductive health with different doses, exposure routes, and in vivo models among studies included. Detailed information on all studies is presented in Table S3.

Animal Model	Phthalate(s) Used	Exposure (Dose, ROA, Duration)	Main Effects	Reference
Four-week-old female ICR mice	DEHP	0, 500, 1000, 1500 mg/kg/day by gavage for 30 days	- ↑ autophagy, - Apoptosis, - Inflammation in ovarian granulosa cells and follicular atresia, - Dose-dependent effects observed.	[54]
Four-week-old female Swiss Albino mice	DEP	1500 mg/kg/day by gavage for 56 days	- Irregular ovarian shape, - ↑ ovarian size, - ↓ secondary follicle dimensions - hormonal disruption (↑ FSH/LH and ↓ estrogen), - No significant change in ovarian weight relative to body weight (GSI).	[55]
Twenty-eight-day-old female CD-1 mice	DBP	10, 100, 1000 mg/kg/day orally for 10, 20, or 30 days	Time- and dose-dependent changes in DNA methyltransferase activity in uterine nuclear extracts with the following: - ↑ activity at 20 days (100 mg/kg/day) - Significant ↓ at 30 days across all doses. - No significant changes in estrous cyclicity.	[56]
Thirty-three-day-old female CD-1 mice	DEHP, DiNP	0.15, 1.5, 1500 ppm for each, orally for 1 month and 6 months	- Short-term exposure decreased FSH levels. - Long-term exposure: (i) primordial follicles ↑, (ii) FSH levels ↑, (iii) preantral and antral follicles ↓, (iv) LH levels ↓. - Both exposures upregulated pituitary genes (Nr5a1, Cga), - No significant changes observed in LH, progesterone, testosterone, estradiol, ovarian follicle populations (short-term), or ovarian steroidogenic gene expression.	[51]
Six-week-old female CD-1 mice	DEHP, DiNP, (DEHP, DiNP, BBP, DBP, DiBP, DEP) mixture	0.15, 1.5, and 1500 ppm, dietary exposure via rodent chow for 11 months	DEHP and Mix exposure at 0.15 and 1500 ppm for 3–5 months: - ↑ time in estrus - time in metestrus/diestrus.	[52]
Newborn male ICR mice	DEHP	30, 500 mg/kg/day orally from birth to postnatal day 21	- Testicular damage (thinning, incomplete tissue, reduced spermatogenic cells), - ↑ apoptosis, - ↓ sperm count and motility, - ↓ serum hormones (GnRH, FSH, LH, T, E2), - Inhibition of Wnt/β-catenin pathway.	[53]
Eight-week-old male C57BL/6N mice	DBP	0, 10, 100 mg/kg/day by gavage for 5 weeks	- ↓ testicular testosterone, - ↑ oxidative stress, - ↑ steroidogenic enzyme expression, - Minor changes in testicular markers and seminiferous tubule morphology, - ↑ in LHR expression at higher doses, - Non-significant upward trends in progesterone/androstenedione levels.	[54]
Eight- to nine-week-old male C57BL/6J mice	DBP, DEHP, (DBP + DEHP) mixture	2.5 mg/kg/day for each group by subcutaneous osmotic pumps for 40 days	- ↓ sperm concentration, - ↑ abnormal sperm morphology (head, neck, tail defects, including decapitated sperm), - ↓ PKA phosphorylation. - No significant differences in sperm motility or tyrosine phosphorylation across groups.	[55]
Male Sprague-Dawley rats	DEHP, DBP, BBP mixture	16 mg/kg/day orally for 91 days	- Testicular damage, - ↓ anogenital distance, - ↑ abnormal sperm rates (decapitation, broken tails, abnormal heads), - disrupted steroidogenesis and spermatogenesis, - ↓ serum testosterone, LH, FSH, dehydroepiandrosterone, androstenedione, dihydrotestosterone, and estrone levels. - Although testes weight and relative testes weight tended to decrease in the MPEs group, these changes were not statistically significant. - Upregulated PIWIL1, StAR, CYP11A1, and CYP17A1. - Downregulated PIWIL2, 17β-HSD, and CYP19A1.	[47]
Twenty-eight-day-old male Fischer CDF344 rats	MEHP	700 mg/kg single dose by gavage once	Increased MHC-II+ peritubular macrophages (CD68+) and PLZF+ spermatogonia, indicating immune activation and potential spermatogenesis disruption.	[56]

### 3.3. In Vitro Studies

To further understand the cellular basis of phthalate toxicity, we focused on studies that employed human cell lines and samples (Table 4). These studies encompass a range of cell lines and experimental models, including human granulosa cells (n = 5), adrenocortical carcinoma cells (n = 1), prostate cells (n = 1), human ovarian tissue (n = 1), and sperm cells (n = 2). The studies also employed different concentrations of various phthalates in an attempt to analyze the different mechanisms through which these compounds impact the reproductive health in humans.

Exposure to MEHP, the main metabolite of DEHP, was shown to have significant impacts on various cellular functions. In the KGN cell line, exposure to MEHP led to a dose-dependent reduction in cell viability, with notable effects occurring at concentrations of 200 µM or more (after an exposure of 24 h). The exposure also led to induced apoptosis through the upregulation of pro-apoptotic proteins (Bax, Cleaved-Caspase-3) alongside the downregulation of the anti-apoptotic protein Bcl-2. Furthermore, it increased the phosphorylation of IκBα, which activated the NF-κB signaling pathway, resulting in elevated expression of pro-inflammatory cytokines (TNF-α, IL-6, IL-1β). MEHP also increased levels of autophagic markers (Beclin-1, Atg5, p62, LC3-II) and triggered oxidative stress (increased ROS, decreased GSH and SOD), indicating its multifaceted disruption of granulosa cell function [45,54]. Additionally, MEHP led to enhanced follicular degeneration and a decrease in growing follicles in human ovarian tissue cultures. The mechanistic investigations in ovarian cell lines indicated disruption of the pathways involving the downregulation of both CTNNB1 and YWHAE and potential alterations in cytoskeletal organization (upregulation of CSRP2), which adversely affects the survival and development of ovarian follicles [57].

Beyond the ovarian cell toxicity, DBP exposure in the PNT1A cell line (derived from human prostate tissue) increased oxidative stress, altered redox homeostasis, and disrupted reproductive hormone signaling by interfering with both estrogen receptor alpha (ERα) and androgen receptor (AR) activity [58]. DBP caused prolonged nuclear translocation of ERα, in contrast to the temporary activation observed with endogenous estradiol (E2), resulting in altered transcriptional regulation. When DBP is paired with testosterone (T) and E2, it maintains AR activation within the nucleus, resembling conditions of hyperandrogenism. These dysregulations enhanced cellular viability and migration, raising the potential of malignant transformation in prostate cells [58]. Similarly, in the human adrenocortical carcinoma cell line H295R, DBP significantly lowered testosterone and androstenedione levels, with a more substantial decrease at higher concentrations, particularly under dbcAMP (synthetic analog of cyclic AMP) stimulated conditions [59]. Finally, DBP exposure resulted in decreased progesterone levels during dbcAMP stimulation, downregulated steroidogenic enzyme levels (CYP11A1, HSD3β2), and elevated superoxide generation, indicative of oxidative stress [59].

DEHP and DnOP were found to accumulate significantly in human sperm cells, in comparison to other phthalates. The results of PAE concentration in sperm were measured using liquid chromatography-mass spectrometry. The observed accumulation is potentially associated with their high lipophilic index (logP) [60]. On top of that, DnOP impaired sperm motility and disrupted the progesterone-mediated acrosome reaction, a critical step for fertilization, by inhibiting phospholipase A2 (PLA2) and ultimately impairing sperm function [60]. In contrast, other work showed that MEHP enhanced sperm penetration ability, hyperactivation, and increased the spontaneous acrosome reaction, potentially due to elevated intracellular calcium levels and tyrosine phosphorylation [61]. These results indicate that the molecular impact of phthalates on the acrosome reaction remains controversial. Furthermore, it remains a critical shortcoming of the field that the in vitro effects of phthalates are only evaluated upon short term exposure for a few hours to days and only in cancer cell lines. Due to the plethora of major metabolic, morphological, and functional differences between cell lines and primary cells, the physiological relevance of the reported in vitro findings remains unclear and it thus remains difficult to draw reliable translational conclusions.

**Table 4:** Overview of the main toxicological effects of phthalates on reproductive health, as demonstrated by the in vitro studies included.

Cell Line(s)/Sample(s)	Human Tissue Type	Phthalate(s) Used	Concentration and Time of Exposure	Main Effects	Reference
KGN	Ovarian Granulosa Tumor	MEHP	0-800 μM for 24 h	- ↓ cell viability dose-dependently, - ↑ apoptosis, - Induced autophagy - Activated NF-κB signaling.	[48]
KGN	Ovarian Granulosa Tumor	MEHP	0–200 μM for 24 h	- ↓ cell viability dose-dependently, - Induced autophagy, - Disrupted autophagic flux - ↑ oxidative stress.	[39]
KGN	Ovarian Granulosa Tumor	DEHP	0.01, 0.1, 1, 10 µM for 24 h	- Impaired cell viability, - ↑ oxidative stress, - ↓ mitochondrial DNA copy numbers and membrane potential, - Upregulated HDAC3 expression at mRNA and protein levels.	[62]
H295R	Adrenocortical Cancer	DBP, MBP	0, 1, 10, 100, 500 µM each for 48 h	DBP: - ↓ testosterone, androstenedione, and progesterone (under dbcAMP stimulation), - ↓ CYP11A1 and HSD3β2 enzyme levels, - ↑ oxidative stress. MBP: - ↓ testosterone and androstenedione (under dbcAMP stimulation) and CYP17A1 enzyme levels.	[59]
A2780, OVCAR5	Ovarian Adenocarcinoma	DEHP	0, 1, 10, 100 μg/mL (time not stated)	In A2780: - Upregulated DUB genes (USP12, ATXN3L, COPS5, USP49), disrupting cell cycle regulation. In OVCAR5: - Upregulated USP49 and downregulated USP34, impairing DNA damage repair and promoting cell death.	[63]
PNT1A	Prostate	DBP	10^−12^ M to 10^−6^ M for 30 min, 2 h, 4 h	- ↑ prostate cell viability, - Altered ERα/AR expression and nuclear translocation, - ↑ oxidative stress, - Promoted cell migration.	[58]
Granulosa Cells	Ovary	DEP, DEHP, DBP, DiNP, DiBP, BBzP mixture	1, 10, 100, 500 μg/mL 24–48 h pre-hCG treatment, followed by 0, 6, 12, 24, or 36 h post-hCG	- ↓ progesterone production at 100 and 500 μg/mL, - Altered PGR expression, - Downregulated steroidogenic genes, - ↓ cAMP and PKA activity at 500 μg/mL, - Disrupted ovulatory mediators.	[64]
Human Ovarian Tissue; KGN, COV434, PA-1, Ovarian Primary Cells	Ovarian Granulosa Tumor: KGN, COV434 Ovarian Germ Cell Tumor: PA-1 Ovary: Human Ovarian Primary Cells	MEHP	0.1X, 1X, 10X, 100X, 1000X (2.05 nM–20.51 mM) for 7 days	In ovarian tissue: - ↑ follicular degeneration, - ↓ growing follicles, - Upregulated CSRP2 in all cell types, - Downregulated CTNNB1 and YWHAE in primary cells, - Disrupted cytoskeletal organization and Hippo signaling. - No significant change in steroid hormones, cytokines, chemokines, and cell viability.	[57]
Human Sperm Cells	Semen	DBP, DnOP, and (DMP, DEP, BBP, DEHP, DnOP) mixture	DBP, DnOP: from 1 ng/mL to 1000 ng/mL for 2 h Mixture: 100 ng/mL for 2 h	- DEHP and DnOP accumulation in sperm cells, - DnOP reduced sperm motility and progesterone-mediated acrosome reaction (AR), - DBP and DnOP inhibited PLA2 - DnOP impaired A23187-induced AR. - AA addition did not restore AR in DBP-exposed sperm.	[60]
Granulosa Cells	Ovary	DEHP	50, 100, 200 μM for 24 h and 48 h	- ↓ cell viability, - ↑ apoptosis, - Induced mitochondrial fission, - ↑ ROS, - ↓ MMP, estradiol-17β and progesterone production.	[65]
Human Sperm Cells	Semen	DEHP, MEHP, (DEHP, MEHP) mixtures	DEHP: 20 nM, 200 nM, 2 µM, 4 µM, 8 µM, MEHP: 1 nM, 10 nM, 100 nM, 1 µM, 20 µM for 1, 2, 4 h	- Enhanced sperm penetration ability, - Hyperactivation, - Spontaneous AR, - Enhanced intracellular calcium levels, and tyrosine phosphorylation, - No change in sperm viability, membrane integrity, motility, ROS levels, or mitochondrial activity.	[61]

**Figure 1:**
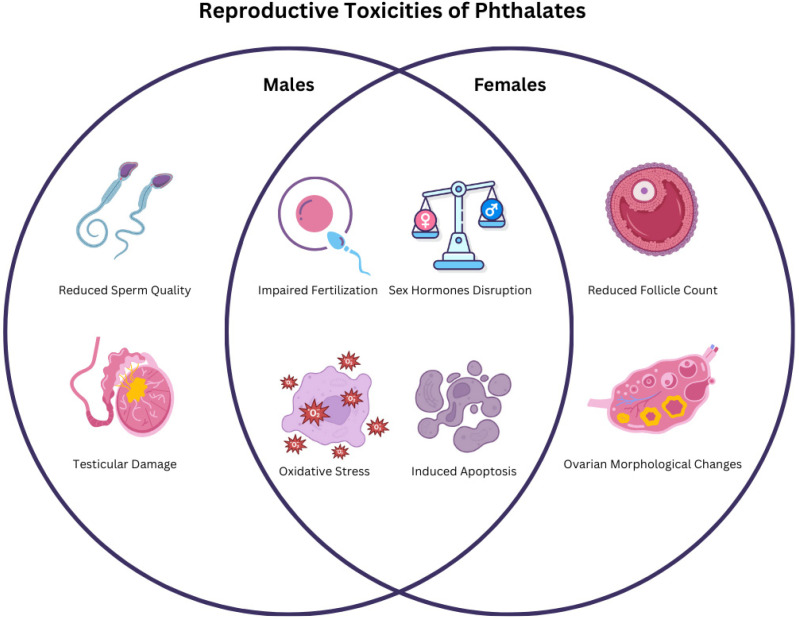
Venn diagram summarizing the main reproductive toxicity outcomes of phthalates in males and females as described in the various studies.

Following the analysis of data gathered from both in vivo and in vitro studies, the main reproductive toxicities identified by the research are illustrated in the schematic representation below (Figure 1).

### 3.4. Epidemiological Studies

Besides in vitro and animal study, our search strategy included various epidemiological studies that covered a wide variety of population ages and geographical regions (Table 5 and Table S4). Out of 30 studies, 17 were conducted in China, which is considered the largest consumer of phthalates in the world [62]. These studies established a positive correlation between phthalate exposure and increased inflammatory cytokines in the follicular fluid (FF) [63], lower levels of serum testosterone (T) and estradiol (E2) [64], reduced sperm quality [65,66], and decreased oocyte count and development [67]. Urinary phthalate metabolite levels were also reported to be significantly higher in Saudi Arabia and Jordan, which coincides with the widespread usage of phthalate-containing products in the Middle East [68,69].

Exposure to phthalates can vary significantly across different occupations and lifestyles, with certain professions and habitual product use posing higher risks. For example, individuals working in farming, dentistry, and artisanal work had a higher risk of infertility, with an odds ratio (OR) of 2.766 (95% CI 1.236–6.185), which was linked to DnBP and DEHP exposure [70]. Moreover, the frequent use of skin beauty products (e.g., fragrances, skin and eye makeup, sunscreen, nail polish), or heating plastic containers in the microwave was linked to higher phthalates exposure, raising the likelihood of infertility and other reproductive problems [69,70].

Phthalates have been repeatedly associated with disruptions in hormone regulation, with various studies underlining their effects on reproductive hormones in both sexes. One such study indicated that MEP and MEOHP significantly decreased cortisol (COR) and corticosterone (CORT) levels in women with diminished ovarian reserve, resulting in a lower number of retrieved oocytes [71]. Furthermore, in men, phthalate mixtures were found to have an inverse correlation with serum T and E2 levels while showing a positive correlation with urinary T and E2 levels, implying that phthalates may affect hormonal metabolism and excretion [64]. Nonlinear effects of phthalates were also observed on ovarian reserve indicators. For instance, MCMHP notably decreased follicle-stimulating hormone (FSH), while MEP significantly increased it solely in the third quartile (moderate levels). Likewise, a positive correlation was found between MEHHP and anti-Müllerian hormone (AMH) exclusively in the fourth quartile (high levels), whereas MiBP exhibited a significant negative correlation with it in the third quartile [72]. These results underscore the complex and dose-dependent hormonal disruptions induced by phthalates, which contribute to impaired reproductive health in humans.

Numerous studies on human cohorts focused on phthalate exposure in adults (n = 26), while others examined exposure during childhood and adolescence (n = 4). The respective findings demonstrate how phthalates can influence reproductive health in different ages. For instance, high exposure to anti-androgenic phthalates (AAPs), such as DEHP, DiNP, MBzP, MnBP, and MiBP, has been associated with delayed puberty in boys, with delays of 8 to 14 months in pubarche and 5.4 to 8.3 months in testicular development [73]. In girls, continuous exposure to phthalates caused an early pubertal onset [74]. Notably, adolescent women had higher levels of phthalate metabolites, especially MBP and MiBP, in the follicular fluid than adults, which is potentially linked to the 248 differentially expressed genes (DEGs) in cumulus cells involved in ovarian cell maturation during adolescence [75]. Interestingly, another study showed a positive correlation between MBP, MEOHP, and the molar sum of phthalate metabolites (∑PAE) levels with the antral follicle count (AFC) in women over 35 years. However, the same study showed an inverse association in younger women, suggesting that age can play a significant role in modulating the effects of phthalate exposure on reproductive health [76].

Longitudinal studies can also provide valuable insights into the long-term effects of early-life exposure to phthalates and how they can affect reproductive health. Higher urinary MiBP levels during early puberty correlated with a trend toward declining semen quality. Additionally, higher urinary concentrations of the molar sum of DiNP metabolites (∑DiNP) during late puberty were strongly associated with reduced semen quality in adulthood [77]. This suggests that the late puberty period may be a crucial time for phthalate exposure, leading to long-term effects on semen quality. Combined, these findings emphasize the various ways in which these substances can interfere with the early stages of life, pointing out the importance of considering the different developmental periods when evaluating the reproductive toxicities of phthalates.

## 4. Discussion

### 4.1. Synthesis of the Key Reproductive Toxicities of Phthalates

The combined findings extracted from the in vivo, in vitro, and epidemiological studies highlight the varied reproductive toxic effects of phthalates in both male and female individuals. These effects can occur through various pathways such as the following: (i) hormonal disruption, (ii) oxidative stress, (iii) induction of apoptotic and inflammatory pathways, and (iv) structural damage to reproductive organs.

Research conducted with animal models (Table 3 and Table S3) showed that phthalates like DEHP, BBP, and DBP, as well as their various mixtures, resulted in dose-dependent testicular damage. This becomes evident by the marked decrease in testicular weight, degeneration of the seminiferous tubules, and a compromised blood–testis barrier (BTB) due to the downregulation of tight junction proteins (ZO-1 and CX-43) and adhesion molecules (N-cadherin). These structural alterations are linked to impaired spermatogenesis, along with the downregulation of spermatogenic proteins (PIWI), as mirrored in reduced sperm count, lower motility, and increased morphological defects. Moreover, a decline in serum testosterone levels has been observed, which is attributed to the inhibition of steroidogenic enzymes (CYP11A1, 17β-HSD) and the alterations in Leydig cell activity. In female rodents, ovarian toxicity is characterized by a decrease in ovarian weight and serum estradiol levels, follicular atresia, and a reduced ovarian reserve. This is achieved through various mechanisms, such as granulosa cell pyroptosis triggered by the activation of the NLRP3 inflammasome through the NF-κB signaling pathway.

In vitro studies strengthen these conclusions (Table 4), indicating that phthalate metabolites such as MEHP can adversely impact granulosa cell viability by triggering oxidative stress, apoptosis, and autophagy. Additionally, they disrupt steroidogenesis by decreasing the expression of StAR and CYP19A1, while also modifying cytoskeletal arrangement and Hippo signaling, ultimately compromising follicle survival. Similarly, in prostate cells, phthalates increased oxidative stress and disrupted reproductive hormone signaling by affecting both estrogen and androgen receptors, whereas in sperm cells, phthalates with a high lipophilic index, such as DnOP, demonstrated greater accumulation and inhibited phospholipase A2, which is essential for the acrosome reactions necessary for fertilization. Both in vitro and in vivo studies provide information regarding the impact of phthalates in cell function (e.g., apoptosis and hormone expression levels) or morphology. However, the studies provide different types of information such as protein expression levels in cells (Table 4) and cell viability, while in vivo experiments (Table 3) focus more on the interplay observed between cells in the same issue and the various signaling pathways between cells.

Moreover, epidemiological evidence supports the real-world implications of these observations (Table 5 and Table S4), associating phthalate exposure with clinical outcomes such as infertility, hormonal imbalance, and reduced ovarian reserve and sperm quality. Dose-dependent hormonal disruptions are observable in both sexes; urinary phthalate metabolites inversely correlate with serum testosterone and estradiol, while also altering gonadotropin levels and exhibiting nonlinear effects on FSH and AMH. Moreover, the timing of exposure during the developmental stages is critical, since exposure during prepuberty can lead to a delay in male puberty, while prolonged exposure hastens its onset in females. Phthalate exposure in males during early and late puberty has been associated with a decline in sperm quality in adulthood. For females, exposure has been found to decrease antral follicle count in women under 35, although older women exhibited the opposite effect. The epidemiological data emphasize how age profoundly influences reproductive risks of phthalate exposure. Additionally, occupational and lifestyle factors, including cosmetic usage, heating plastic containers, and agricultural work, increase exposure risks, especially in regions with high consumption rates like China and the Middle East.

### 4.2. Strengths and Limitations of the Systematic Review

This systematic review offers a thorough synthesis of the existing evidence (January 2020–June 2024) concerning the reproductive toxicities of phthalates, combining findings from in vivo, in vitro, and epidemiological studies. The inclusion of various study designs, ranging from mechanistic explorations in human cell lines to population-based cohort research, strengthens the overall conclusions. Following PRISMA guidelines enhances methodological clarity while the introduction of predetermined eligibility criteria allows the researchers to focus on specific aspects in their analysis while avoiding dual entries or incorporating gray literature. Furthermore, by addressing both male and female reproductive outcomes, the review provides a well-rounded view of phthalate-induced reproductive toxicity, emphasizing dose-dependent effects and various mechanisms through which phthalates disrupt hormonal balance, cellular function, and reproductive outcomes.

Nonetheless, there are limitations regarding the scope of this review since the exclusion of studies involving co-exposure to other chemical groups limits the understanding of the way phthalates interact with other environmental pollutants and how they impact human reproductive health. Furthermore, the varying methodologies, endpoints, and levels of phthalate exposure in the studies make it challenging to formulate direct comparisons. For instance, in vivo studies utilized a wide range of doses, different exposure durations, and various rodent strains, which may affect the toxicity results. Additionally, in vitro studies offered valuable insights into the molecular mechanisms of phthalate toxicities, yet their small number (n = 11), combined with the use of concentrations that exceed normal environmental exposure levels, raises concerns about the diversity of mechanistic findings and their physiological relevance. While epidemiological studies offer valuable real-world insights, many do not adequately address confounding factors (such as co-exposure to other chemicals and lifestyle variables), which may alter or distort the associations reported. The prevalence of cross-sectional designs in human studies also constrains causal conclusions, and longitudinal data on early-life phthalate exposure and long-term reproductive outcomes remains limited.

Unlike other persistent organic pollutants, phthalates typically have short half-lives (usually less than 24 h in both humans and rodents) [78], indicating that their effects may be contingent upon repeated or chronic exposure. However, most studies did not evaluate the duration of exposure or accumulation, instead concentrating on single-time measurements. This oversight in study design complicates the assessment of long-term reproductive toxicity of phthalates. Finally, this review does not pool effect sizes or formally test dose–response trends through meta-analyses. Broadening the publication window beyond 2020–2024 may be necessary in future work to attain sufficient sample size and reduce the risk of introducing bias in the analysis.

### 4.3. Research Gaps and Future Directions

Despite notable advances in understanding the reproductive toxicities associated with phthalates, significant gaps in research remain. Firstly, although DiNP and DiDP are prohibited in toys, they are still commonly utilized as substitutes for DEHP in the manufacturing of PVC products despite insufficient toxicological assessment [16]. For instance, some studies included in this review correlate DiNP exposure to delayed male puberty and diminished sperm quality, which are serious reproductive risks comparable with the impact of DEHP. However, the mechanisms behind these effects are still poorly understood, and there is a lack of comprehensive epidemiological data. This complicates efforts to align industrial reliance on these substitutes with protections for reproductive health.

Moreover, most studies evaluate exposure in the general population and more focused research into particularly exposed or vulnerable populations, such as children, adolescents, and occupationally exposed groups (e.g., workers in PVC manufacturing or agriculture), which promises to provide important insights into phthalate effects in at-risk groups. Exposure to phthalates has been associated with alterations in puberty onset in both males and females, yet the specific molecular mechanisms driving these sex-dependent effects remain unclear [73,74]. There is an urgent need for longitudinal studies to determine how exposure during crucial developmental stages, such as the pre-, early, and late pubertal periods, could affect reproductive health across a person’s lifespan. In addition, groups that experience higher cumulative exposure levels through their occupation are poorly researched despite their significantly increased risk, which highlights a major gap in both academic study and protective policy measures.

Critically, research designs often utilize high-dose, short-duration exposures that do not accurately represent real-life exposure circumstances (chronic, low-dose). For example, studies examining the impact of DEHP on sperm quality and ovarian reserves usually employed doses much higher than what individuals are normally exposed to, which might not provide an accurate assessment of its effects. Similarly, mechanistic investigations should consider the use of emerging organotypic and microphysiological human culture models instead of cell lines to increase translational relevance and align with societal and regulatory objectives [79]

Ultimately, enhancing structure–activity relationship (SAR) research and utilizing computational tools could transform phthalate risk management. The current knowledge regarding the mechanism with which specific chemical structures influence toxicity (e.g., the length of ester chains or degree of aromaticity) requires more attention. Machine learning (ML) algorithms, developed using extensive toxicological data, could forecast interactions between phthalates and receptors (like estrogen or androgen receptors) to identify potential dangers. For example, ML-driven virtual screening could reveal plasticizers that retain their functional properties while minimizing endocrine disruption. Furthermore, recent work has shown that ML models, such as logistic regression, artificial neural networks, XGBoost, and Bayesian networks, can predict disease risks based on a combination of demographic data, laboratory data (including plasticizer metabolite levels in human urine), and lifestyle habits [80]. These models identified associations between specific phthalates and musculoskeletal conditions such as arthritis and osteoporosis, demonstrating ML’s potential to uncover exposure-related health effects [80]. Using pharmacological risk assessments as a blueprint [81], the combined use of these innovative in silico tools with reliable experimental validation assays can pave the way for earlier hazard identification and a faster transition toward the use of non-toxic materials.

## 5. Conclusions

The review compiles evidence from 90 studies to underscore the significant reproductive toxicity of phthalates in both males and females. Research conducted both in vivo and in vitro indicates that phthalates impair spermatogenesis, lower ovarian reserve, and disrupt hormonal balance via oxidative stress, apoptosis, and inflammatory mechanisms. Epidemiological research further links phthalate exposure to infertility, altered hormone levels, and developmental delays during puberty. Regulatory actions have limited the use of high-risk phthalates like DEHP, while compounds such as DiNP and DiDP are being increasingly utilized in PVC production due to their assumed lower toxicity. However, there is emerging evidence suggesting that these compounds also pose reproductive risks, necessitating urgent toxicological assessment. The key limitations in toxicological assessment include inconsistent exposure assessments and insufficient data on early life exposures or long-term effects. Future research needs to prioritize vulnerable populations, realistic exposure scenarios, and computational approaches to evaluate the toxicity of phthalates and develop safer alternatives. Addressing these gaps will enhance regulatory frameworks and reduce the worldwide impact of phthalate-induced reproductive toxicity.

## Figures and Tables

**Table 1:** Parent phthalates according to their molecular weight, with their primary and secondary metabolites listed.

	Parent Phthalate	Primary Metabolite	Secondary Metabolite
**High Molecular Weight (HMW) Phthalates**	DEHP	MEHP	MEHHP MEOHP MECPP
	DiNP	MiNP	MHiNP MOiNP MCiNP
	DiDP	MiDP	MHiDP MOiDP MCiDP
	DnOP	MnOP	MCPP
**Low Molecular Weight (LMW) Phthalates**	BBP	MBzP	-
	DMP	MMP	-
	DEP	MEP	-
	DBP	MnBP	MHnBP
	DiBP	MiBP	MHiBP

Legend: DEHP: Di(2-ethylhexyl) phthalate, DiNP: Diisononyl phthalate, DiDP: Diisodecyl phthalate, DnOP: Di-n-octyl phthalate, BBP: Benzyl butyl phthalate, DMP: Dimethyl phthalate, DEP: Diethyl phthalate, DBP: Dibutyl phthalate, DiBP: Diisobutyl phthalate, MEHP: Mono(2-ethylhexyl) phthalate, MiNP: Monoisononyl phthalate, MiDP: Monoisodecyl phthalate, MnOP: Mono-n-octyl phthalate, MBzP: Monobenzyl phthalate, MMP: Monomethyl phthalate, MEP: Monoethyl phthalate, MnBP: Monobutyl phthalate, MiBP: Monoisobutyl phthalate, MEHHP: Mono(2-ethyl-5-hydroxyhexyl) phthalate, MEOHP: Mono(2-ethyl-5-oxohexyl) phthalate, MECPP: Mono(2-ethyl-5-carboxypentyl) phthalate, MHiNP: Mono(hydroxyisononyl) phthalate, MOiNP: Mono(oxoisononyl) phthalate, MCiNP: Mono(carboxyisooctyl) phthalate, MHiDP: Mono(hydroxyisodecyl) phthalate, MOiDP: Mono(oxoisodecyl) phthalate, MCiDP: Mono(carboxyisononyl) phthalate, MCPP: Mono(3-carboxypropyl) phthalate, MHnBP: Mono(hydroxybutyl) phthalate, MHiBP: Mono(hydroxyisobutyl) phthalate.

**Table 2:** Overview of commonly used phthalates.

Phthalate Name	Abbreviation	CAS Registry Number	Molecular Formula	Chemical Structure	Molecular Weight (g/mol)
Di-(2-ethylhexyl) phthalate	DEHP	117-81-7	C_24_H_38_O_4_	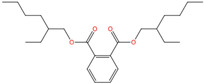	390.56
Di-n-butyl phthalate	DBP	84-74-2	C_16_H_22_O_4_	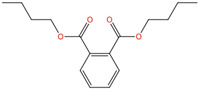	278.34
Benzyl butyl phthalate	BBP	85-68-7	C_19_H_20_O_4_	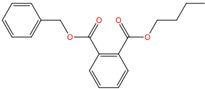	312.36
Diethyl phthalate	DEP	84-66-2	C_12_H_14_O_4_	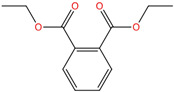	222.24
Diisobutyl phthalate	DiBP	84-69-5	C_16_H_22_O4	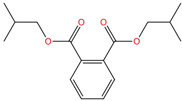	278.34
Diisononyl phthalate	DiNP	28553-12-0	C_26_H_42_O_4_	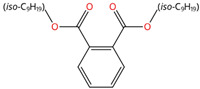	418.61
Diisodecyl phthalate	DiDP	89-16-7	C_28_H_46_O_4_	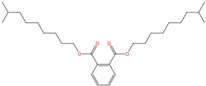	446.66
Dimethyl phthalate	DMP	131-11-3	C_10_H_10_O_4_	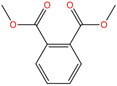	194.18
Di-n-octyl phthalate	DnOP	117-84-0	C_24_H_38_O_4_	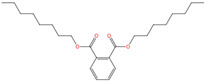	390.56
Mono-(2-ethylhexyl) phthalate	MEHP	4376-20-9	C_16_H_22_O_4_	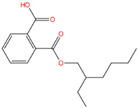	278.34
Mono-2-ethyl-5-hydroxyhexyl phthalate	MEHHP	40321-99-1	C_16_H_22_O_5_	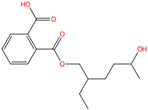	294.34
Mono-n-butyl phthalate	MnBP	131-70-4	C_12_H_14_O_4_	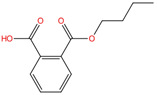	222.24
Mono-benzyl phthalate	MBzP	2528-16-7	C_15_H_12_O_4_	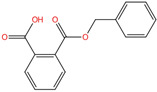	256.25
Mono-n-octyl phthalate	MnOP	5393-19-1	C_16_H_22_O_4_	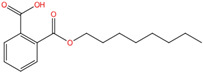	278.34
Mono-ethyl phthalate	MEP	2306-33-4	C_10_H_10_O_4_	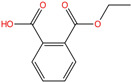	194.18
Mono-isobutyl phthalate	MiBP	30833-53-5	C_12_H_14_O_4_	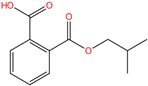	222.24
Mono-methyl phthalate	MMP	4376-18-5	C_9_H_8_O_4_	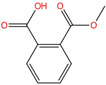	180.16
Mono-isopropyl phthalate	MiPrP	35118-50-4	C_11_H_12_O_4_	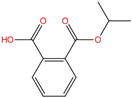	208.21
Mono-isononyl phthalate	MiNP	106610-61-1	C_17_H_24_O_4_	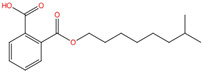	292.37
Mono(2-ethyl-5-carboxypentyl) phthalate	MECPP	40809-41-4	C_16_H_20_O_6_	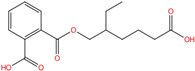	308.33
Mono(2-ethyl-5-oxohexyl) phthalate	MEOHP	40321-98-0	C_16_H_20_O_5_	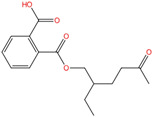	292.33
Mono [2-(carboxymethyl)hexyl phthalate	MCMHP	82975-93-7	C_16_H_20_O_6_	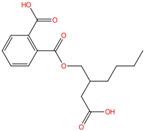	308.33
Mono-3-carboxypropyl phthalate	MCPP	66851-46-5	C_12_H_12_O_6_	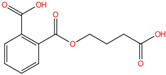	252.22

**Table 5:** Overview of the epidemiological studies on phthalate exposure and its associations with reproductive health outcomes, including number of studies, study design, population, and phthalates examined. Detailed information is presented in Table S4.

Study Design	No. of Studies	Population	Phthalate/Phthalate Metabolite Examined	Association/Examined Outcome
C-S	16	- Women undergoing IVF - Women with diminished ovarian reserve - Fertile/healthy women - Infertile women - Fertile/healthy men - Infertile men - Adolescents	MMP, MEP, MBP, MBzP, MEHP, MEHHP, MEOHP, MOP, MECPP, MCMHP, MCPP, MiBP, MnBP, DEHP, DEP, DBP, DnOP	- Male fertility - Female infertility - Inflammatory cytokines in the follicular fluid - Antral follicle count - Reproductive hormone levels - Diminished ovarian reserve - Semen quality - Menstrual cycle characteristics - Ovarian reserve - Spermatogenesis-related miRNA106a levels - Follicular fluid anti-Müllerian hormone levels
C-C	7	- Fertile/healthy women - Infertile women - Fertile/healthy men - Infertile men - Women with polycystic ovaries - Women with polycystic ovary syndrome - Women with endometriosis - Women with premature ovarian failure - Women with endometrial polyp	DMP, MMP, DEP, MEP, DBP, MBP, BBzP, MBzP, DEHP, MEHP, MEHHP, MEOHP, MnBP, MiBP, MECPP	- Women fertility - Semen quality - Polycystic ovary - Polycystic ovary syndrome - Endometriosis - Premature ovarian failure - Endometrial polyp
C	7	- Children (boys and girls) - Adolescent men - Women undergoing IVF - Men trying to conceive	DEHP, DINP, DiNP, DiDP, MBzP, MnBP, MiBP, MCPP, MECPP, MEHP, MEHHP, MEOHP, MEP, MCOMHP, MCOMOP, MMP, MBP, MCMHP	- Pubertal onset - Reproductive hormone levels - Semen quality - Sperm epigenetic aging - Follicular fluid extracellular vesicle microRNAs (EV-miRNAs) expression - Oocyte yield and quality

Legend: C: cohort, C-S: cross-sectional, C-C: Case–Control, IVF: in vitro fertilization.

## Supplementary Materials

The following supporting information can be downloaded at: https://www.mdpi.com/article/10.3390/ijms26188761/s1.

## Abbreviations

**AAPs**	Anti-Androgenic Phthalates
**AMH**	Anti-Müllerian Hormone
**AR**	Androgen Receptor
**AFC**	Antral Follicle Count
**BTB**	Blood–Testis Barrier
**CAS**	Chemical Abstracts Service
**CI**	Confidence Interval
**COR**	Cortisol
**CORT**	Corticosterone
**CYP**	Cytochrome P450 enzymes (e.g., CYP11A1, CYP17A1)
**dbcAMP**	Dibutyryl Cyclic AMP
**DEGs**	Differentially Expressed Genes
**DUB**	Deubiquitinating Enzyme
**EDCs**	Endocrine-Disrupting Chemicals
**E2**	Estradiol
**EPA**	U.S. Environmental Protection Agency
**ECHA**	European Chemicals Agency
**ERα**	Estrogen Receptor alpha
**EV-miRNAs**	Extracellular Vesicle microRNAs
**FF**	Follicular Fluid
**FSH**	Follicle-Stimulating Hormone
**GCs**	Granulosa Cells
**GSH**	Glutathione
**HMW**	High Molecular Weight
**IVF**	In Vitro Fertilization
**ICSI**	Intracytoplasmic Sperm Injection
**LMW**	Low Molecular Weight
**LH**	Luteinizing Hormone
**logP**	Lipophilicity Index
**MDA**	Malondialdehyde
**ML**	Machine Learning
**NF-κB**	Nuclear Factor kappa B
**NLRP3**	NLR Family Pyrin Domain Containing 3
**OR**	Odds Ratio
**PAEs**	Phthalic Acid Esters
**PCOS**	Polycystic Ovary Syndrome
**PCO**	Polycystic Ovaries
**PLA2**	Phospholipase A2
**PIWI**	P-element Induced WImpy Testis Proteins (e.g., PIWIL1, PIWIL2)
**POF**	Premature Ovarian Failure
**PRISMA**	Preferred Reporting Items for Systematic Reviews and Meta-Analyses
**PVC**	Polyvinyl Chloride
**ROA**	Route of Administration
**ROS**	Reactive Oxygen Species
**SAR**	Structure–Activity Relationship
**SEA**	Sperm Epigenetic Aging
**SOD**	Superoxide Dismutase
**StAR**	Steroidogenic Acute Regulatory Protein
**T**	Testosterone
**T-AOC**	Total Antioxidant Capacity
