# Supplementary material for: A Systematic Literature Review of Reproductive Toxicological Studies on Phthalates
Source: Int J Mol Sci. 2025 Sep 9;26(18):8761. doi: 10.3390/ijms26188761 (PMC12469734; doi:10.3390/ijms26188761)
Supplement: Supplementary file 1 [file ijms-26-08761-s001.zip › ijms-3845688-supplementary.pdf]

Supplementary Material

A Systematic Literature Review of Reproductive Toxicological Studies on Phthalates

Muhammad Moghazy [1,2], Marianthi Papathanasiou [3], Haralampos Tzoupis [4], Konstantinos D. Papavasileiou [1], Chen Xing [5,6,7], Volker M. Lauschke [5,6,7,8], Antreas Afantitis [1,4,9] and Georgia Melagraki [3,*]

[1] Department of Cheminformatics, NovaMechanics MIKE, 185 45 Piraeus, Greece;
[2] Medical School, National and Kapodistrian University of Athens, 115 27 Athens, Greece
[3] Division of Physical Sciences & Applications, Hellenic Military Academy, 16673 Vari, Greece;
[4] Department of Cheminformatics, NovaMechanics Ltd., Nicosia 1070, Cyprus;
[5] Dr. Margarete Fischer-Bosch Institute of Clinical Pharmacology, Auerbachstraße 112, 70376 Stuttgart, Germany
[6] Geschwister-Scholl-Platz, University of Tübingen, 72074 Tübingen, Germany
[7] Department of Physiology and Pharmacology and Center for Molecular Medicine, Karolinska Institute and University Hospital, 171 76 Stockholm, Sweden
[8] Department of Pharmacy, The Second Xiangya Hospital, Central South University, 139 Renmin, Changsha 410011, China
[9] Entelos Institute, Nicosia 2102, Cyprus

Table of Contents

Methodology 2

Table S1: *Detailed description of the eligibility criteria of the systematic review.* 2

Search Strategy 3

Table S2: *PRISMA2020 checklist for systematic reviews.* 3

Figure S1: *PRISMA flow diagram summarizing the number of studies screened, excluded, and included at each stage of the review process.* 7

Results 7

Table S3: *Overview of the main toxicological effects of phthalates on reproductive health across doses, exposure routes, and in vivo models among the selected studies.* 7

Table S4: *Summary of epidemiological studies on phthalate exposure and reproductive health outcomes, including study design, population, phthalates detected, and key findings.* 15

Abbreviations 21

References 23

Methodology

Table S1: *Detailed description of the eligibility criteria of the systematic review.*

Parameters	Inclusion	Exclusion
**Publication date**	January 2020 to June 2024	Out of the time period: 01/2020-06/2024
**Language**	English	Non-English
**Population**	• Human studies:<br>a) *in vitro* human cell lines<br>b) biomonitoring/epidemiological studies<br>• Animal studies: only *in vivo* studies of laboratory animals, specifically: rats and mice	• *In vitro* studies with animal cell lines<br>• *In vivo* studies employing animal models where experimental endpoints lack translational validity for human reproductive health (e.g., zebrafish (*Danio rerio*) and nematodes (*C. elegans*)
**Exposure**	• Studies investigating exposure to phthalates (e.g., DEHP, DBP, BBP, DnOP, DINP, DIDP, etc.) individually or in mixtures	• Studies not involving phthalates<br>• Co-exposure of phthalates and other chemicals
**Outcomes**	Studies reporting on at least one of the following outcomes:<br>• Fertility (e.g., sperm count, sperm quality, follicular count)<br>• Reproductive health (e.g., hormonal disruption, testicular or ovarian function, morphological changes to reproductive organs, puberty onset, etc.)	• Studies that did not directly report on fertility, reproductive health, or reproductive toxicity outcomes. Examples include research on gestational diabetes, fetal development, or pregnancy outcomes.
**Study Design**	• *In vivo* studies in rodents for reproductive toxicological studies<br>• All human studies, either exposure (epidemiological, cohort studies) or *in vitro* human cell lines	• Reviews (systematic, critical etc.) combined or not with Meta-analysis<br>• Case studies and reports<br>• Editorials, letter to editorial<br>• Conference papers, research highlights, books
**Data availability**	Either downloadable dataset or retrievable data from the paper	On request, or not available data

Search Strategy

Query in PubMed (477 results) and Scopus (649 results): ("phthalates" OR "phthalate esters" OR "DEHP" OR "DBP" OR "BBP" OR "DiNP" OR "DiDP") AND ("fertility" OR "reproductive health" OR "infertility" OR "reproductive toxicity" OR "sperm count" OR "ovarian function" OR "hormonal disruption" OR "pregnancy outcomes").

Table S2: *PRISMA2020 checklist for systematic reviews.*

Section and Topic	Item #	Checklist item	Location where item is reported
**TITLE**			
Title	1	Identify the report as a systematic review.	Page 1
**ABSTRACT**			
Abstract	2	See the PRISMA 2020 for Abstracts checklist.	Pages 1-2 (lines 19-34)
**INTRODUCTION**			
Rationale	3	Describe the rationale for the review in the context of existing knowledge.	Page 4 (lines 113-125)
Objectives	4	Provide an explicit statement of the objective(s) or question(s) the review addresses.	Page 5 (lines 136-138)
**METHODS**			
Eligibility criteria	5	Specify the inclusion and exclusion criteria for the review and how studies were grouped for the syntheses.	Section 2.1., (pages 5-7) and Table S1 Supplementary Material (pages 2-3)
Information sources	6	Specify all databases, registers, websites, organisations, reference lists and other sources searched or consulted to identify studies. Specify the date when each source was last searched or consulted.	Section 2.2. (page 9)
Search strategy	7	Present the full search strategies for all databases, registers and websites, including any filters and limits used.	Section 2.2. (page 9)
Selection process	8	Specify the methods used to decide whether a study met the inclusion criteria of the review, including how many reviewers screened each record and each report retrieved, whether they worked independently, and if applicable, details of automation tools used in the process.	Section 2.3. (pages 9-10)
Data collection process	9	Specify the methods used to collect data from reports, including how many reviewers collected data from each report, whether they worked independently, any processes for obtaining or confirming data from study investigators, and if applicable, details of automation tools used in the process.	N/A
Data items	10a	List and define all outcomes for which data were sought. Specify whether all results that were compatible with each outcome domain in each study were sought (e.g. for all measures, time points, analyses), and if not, the methods used to decide which results to collect.	N/A

Section and Topic	Item #	Checklist item	Location where item is reported
	10b	List and define all other variables for which data were sought (e.g. participant and intervention characteristics, funding sources). Describe any assumptions made about any missing or unclear information.	N/A
Study risk of bias assessment	11	Specify the methods used to assess risk of bias in the included studies, including details of the tool(s) used, how many reviewers assessed each study and whether they worked independently, and if applicable, details of automation tools used in the process.	N/A
Effect measures	12	Specify for each outcome the effect measure(s) (e.g. risk ratio, mean difference) used in the synthesis or presentation of results.	N/A
Synthesis methods	13a	Describe the processes used to decide which studies were eligible for each synthesis (e.g. tabulating the study intervention characteristics and comparing against the planned groups for each synthesis (item #5)).	N/A
	13b	Describe any methods required to prepare the data for presentation or synthesis, such as handling of missing summary statistics, or data conversions.	N/A
	13c	Describe any methods used to tabulate or visually display results of individual studies and syntheses.	N/A
	13d	Describe any methods used to synthesize results and provide a rationale for the choice(s). If meta-analysis was performed, describe the model(s), method(s) to identify the presence and extent of statistical heterogeneity, and software package(s) used.	N/A
	13e	Describe any methods used to explore possible causes of heterogeneity among study results (e.g. subgroup analysis, meta-regression).	N/A
	13f	Describe any sensitivity analyses conducted to assess robustness of the synthesized results.	N/A
Reporting bias assessment	14	Describe any methods used to assess risk of bias due to missing results in a synthesis (arising from reporting biases).	N/A
Certainty assessment	15	Describe any methods used to assess certainty (or confidence) in the body of evidence for an outcome.	N/A
**RESULTS**			
Study selection	16a	Describe the results of the search and selection process, from the number of records identified in the search to the number of studies included in the review, ideally using a flow diagram.	Section 3.1. (page 10)
	16b	Cite studies that might appear to meet the inclusion criteria, but which were excluded, and explain why they were excluded.	N/A
Study characteristics	17	Cite each included study and present its characteristics.	Section 3.2. (pages 10-13), Section 3.3. (pages 14-17), Section 3.4.

Section and Topic	Item #	Checklist item	Location where item is reported
			(pages 18-20), Table S3 Supplementary Material (pages 7-15), and Table S4 Supplementary Material (pages 15-21)
Risk of bias in studies	18	Present assessments of risk of bias for each included study.	N/A
Results of individual studies	19	For all outcomes, present, for each study: (a) summary statistics for each group (where appropriate) and (b) an effect estimates and its precision (e.g. confidence/credible interval), ideally using structured tables or plots.	Table 3 (pages 12-13), Table 4 (pages 15-17), Table S3 Supplementary Material (pages 7-15), and Table S4 Supplementary Material (pages 15-21)
Results of syntheses	20a	For each synthesis, briefly summarise the characteristics and risk of bias among contributing studies.	N/A
	20b	Present results of all statistical syntheses conducted. If meta-analysis was done, present for each the summary estimate and its precision (e.g. confidence/credible interval) and measures of statistical heterogeneity. If comparing groups, describe the direction of the effect.	N/A
	20c	Present results of all investigations of possible causes of heterogeneity among study results.	N/A
	20d	Present results of all sensitivity analyses conducted to assess the robustness of the synthesized results.	N/A
Reporting biases	21	Present assessments of risk of bias due to missing results (arising from reporting biases) for each synthesis assessed.	N/A
Certainty of evidence	22	Present assessments of certainty (or confidence) in the body of evidence for each outcome assessed.	N/A
**DISCUSSION**			
Discussion	23a	Provide a general interpretation of the results in the context of other evidence.	Section 4.1. (pages 20-21)
	23b	Discuss any limitations of the evidence included in the review.	Section 4.2. (pages 21-22)
	23c	Discuss any limitations of the review processes used.	Section 4.2. (pages 21-22)
	23d	Discuss implications of the results for practice, policy, and future research.	Section 4.3. (pages 23-24)
**OTHER INFORMATION**			
Registration and protocol	24a	Provide registration information for the review, including register name and registration number, or state that the	Section 2.1. (page 5, lines

Section and Topic	Item #	Checklist item	Location where item is reported
		review was not registered.	153-154)
	24b	Indicate where the review protocol can be accessed, or state that a protocol was not prepared.	Section 2.1. (page 5, lines 153-154)
	24c	Describe and explain any amendments to information provided at registration or in the protocol.	N/A
Support	25	Describe sources of financial or non-financial support for the review, and the role of the funders or sponsors in the review.	Page 25 (lines 571-575)
Competing interests	26	Declare any competing interests of review authors.	Page 25 (lines 577-580)
Availability of data, code and other materials	27	Report which of the following are publicly available and where they can be found template data collection forms; data extracted from included studies; data used for all analyses; analytic code; any other materials used in the review.	N/A

From: Page MJ, McKenzie JE, Bossuyt PM, Boutron I, Hoffmann TC, Mulrow CD, et al. The PRISMA 2020 statement: an updated guideline for reporting systematic reviews. BMJ 2021;372:n71. doi:10.1136/bmj.n71.

Figure S1: *PRISMA flow diagram summarizing the number of studies screened, excluded, and included at each stage of the review process.*

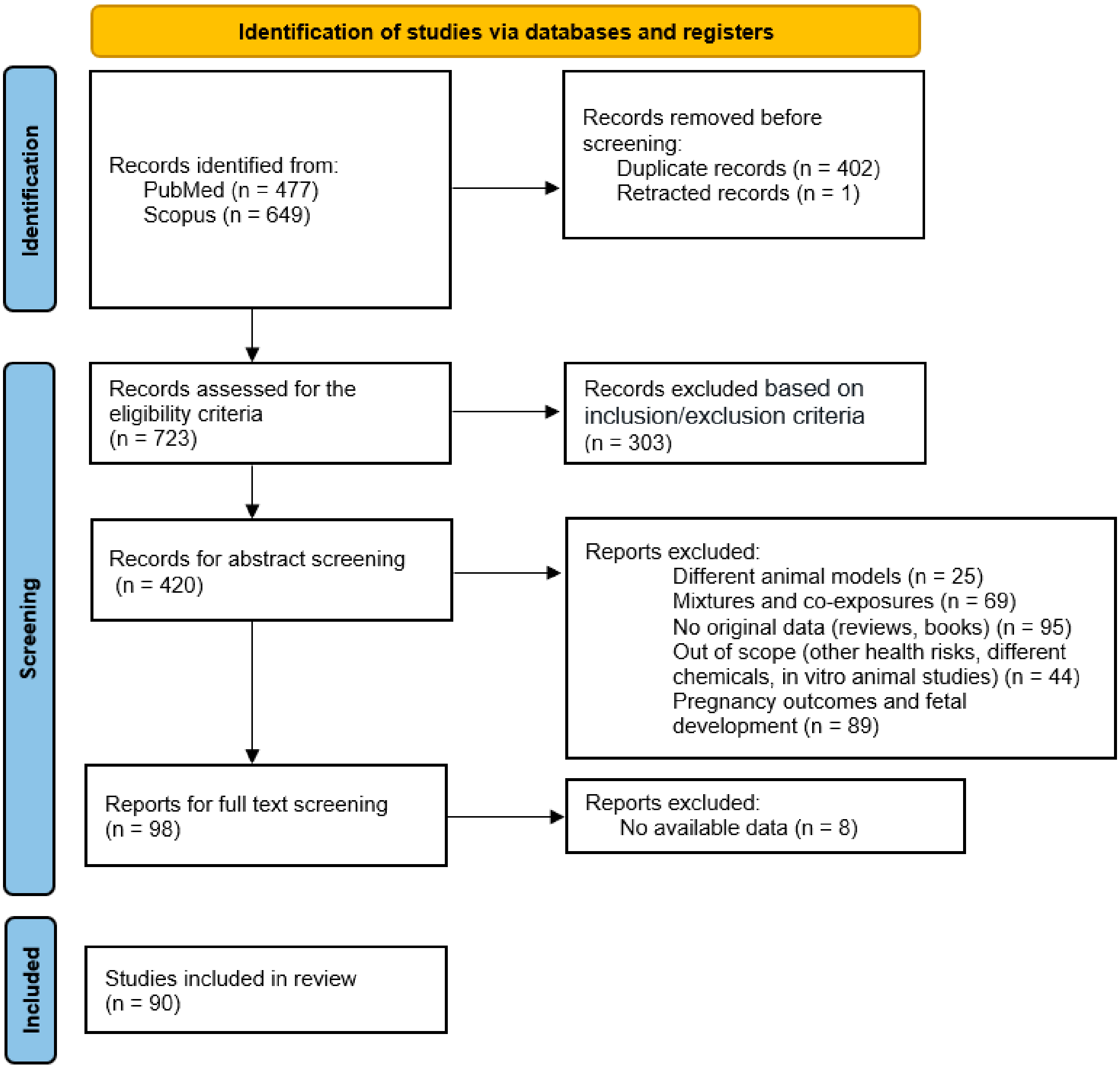

Results

Table S3: *Overview of the main toxicological effects of phthalates on reproductive health across doses, exposure routes, and in vivo models among the selected studies.*

Animal Model	Phthalate(s) Used	Exposure (dose, ROA, duration)	Main Effects	Reference
Four-week-old female ICR mice	DEHP	0, 250, 500, 1000 mg/kg/day by gavage for 30 days	Reduced ovarian weight, follicle counts, and estradiol levels, disrupted estrous cycles, and increased atretic follicles, while dose-dependently upregulating	[1]

			autophagy proteins in granulosa cells.	
Five-week-old male Sprague-Dawley rats	DEHP, DBP, BBP mixture	450 mg/kg/day by gavage for 91 days	Shortened anogenital distance, reduced testes and epididymis weights, and decreased hormone levels (testosterone, LH, FSH, DHEA, androstenedione, estrone, DHT). It caused severe testicular damage, including seminiferous tubule atrophy, arrested spermatogenesis, Leydig cell hyperplasia, and mitochondrial damage.	[2]
Three-week-old male ICR mice	DEHP	500 mg/kg/day by gavage for 4 weeks	Sperm abnormalities, reduced motility, viability, and sperm-egg binding ability, alongside severe testicular damage, including disrupted seminiferous tubules, Sertoli cell degeneration, and compromised blood-testis barrier integrity. Molecular changes included oxidative stress gene upregulation, spermatogenesis gene downregulation, and mitochondrial damage in Leydig and Sertoli cells.	[3]
Ten- to eleven-week-old female Swiss Albino mice	DEHP	500 mg/kg/day by gavage for 4 weeks	Reduced female reproductive tract weight and caused structural abnormalities, including fewer ovarian follicles, disorganized oviduct mucosa, and a smaller uterus with thicker endometrium, alongside reduced secondary and tertiary follicle counts and decreased oviduct epithelial height.	[4]
Three-month-old male Wistar Albino rats	DBP	250, 500 mg/kg, 3 times/week orally for 8 weeks	Dose-dependent reduction in serum testosterone levels, sperm count, viability, and motility, increased oxidative stress (elevated MDA, decreased GSH and CAT), and caused severe degenerative changes in seminiferous tubules, including reduced epithelial thickness and complete cell degeneration at higher doses.	[5]
Seven-week-old female ICR mice	DEHP	20, 40 µg/kg/day from birth to 21 days postpartum via lactation (maternal administration)	Reduced oocyte and antral follicle numbers, disrupted steroidogenesis-related gene expression, decreased 17β-estradiol levels, and increased oxidative stress, DNA damage, and apoptosis in ovarian cells. It also inhibited granulosa cell proliferation, caused spindle	[6]

			abnormalities, and led to chromosome misalignment in oocytes.	
Three-week-old male Sprague-Dawley rats	DEHP	0, 250, 500 mg/kg/day by gavage for 5 weeks	Testicular damage, including reduced organ coefficient, shrunken spermatogenic tubules, decreased Sertoli cells, and lower testosterone levels, particularly at 500 mg/kg. It also disrupted the blood-testis barrier, increased oxidative stress, and triggered ferroptosis.	[7]
Five-week-old male ICR mice	DEHP	0.5, 50, 500 mg/kg by gavage for 5 weeks	Testicular damage, including reduced testis and epididymis weights, damaged seminiferous tubules, decreased sperm count, and elevated germ cell apoptosis, particularly at 500 mg/kg/day. It also increased apoptotic markers and ER stress markers.	[8]
Six- to eight-week-old male ICR mice	DEHP	200 mg/kg/day by gavage for 35 days	Reduced testis weight, serum testosterone levels, sperm motility, count, and daily sperm production, while impairing male fertility and blastocyst formation. It caused histological damage, including loose seminiferous epithelial arrangements and fewer germ cells, alongside increased oxidative stress and apoptosis.	[9]
Thirty-three-day-old female CD-1 mice	DEP, DBP, DIBP, DEHP, BzBP, DiNP mixture	0.15, 1.5, 1500 ppm orally for 1 month (short-term) and 6 months (long-term)	Long-term exposure to 1500 ppm increased primordial follicles but decreased antral follicles and reduced estradiol and LH levels, while short-term exposure at 0.15 ppm increased atretic follicles and decreased Cas3 expression, indicating disrupted folliculogenesis, hormone levels, and apoptosis regulation. Borderline decreases in Gsr and Nr5a1, and mostly unchanged steroidogenesis and cell cycle genes were also observed.	[10]
Newborn male BALB/c mice	DEHP	500 mg/kg single oral dose, administered on day of birth	Reduced testicular weight, delayed gonocyte differentiation, decreased spermatogonia population, and disrupted seminiferous epithelium structure, leading to reduced proliferation, delayed germ cell maturation, and reduced sperm concentration and normal morphology in adulthood.	[11]

Five- to six-month-old male Wistar rats	DEHP	750 mg/kg/day orally for 1, 3, 5, 7, or 9 weeks	Reduced testicular weight, sperm count, motility, viability, and normal morphology, while increasing abnormal sperm morphology and decreasing LH, FSH, testosterone, and serum Zn/Mg levels, alongside progressive testicular damage, including atrophic seminiferous tubules, maturation arrest, and fibrosis.	[12]
Three-week-old male ICR mice	DEHP	500, 1000 mg/kg/day by gavage for 28 days	Testicular damage, including mitochondrial dysfunction and induced mitophagy. Structural damage, such as autophagic vacuoles, swollen mitochondria, disrupted ER, and Sertoli cell degeneration.	[13]
Three-day-old male Sprague-Dawley rats	DEHP	60, 300, 600 mg/kg/day orally and IV for 5 or 21 days starting from PND 3 to PND 7 or PND 23	Dose-dependent testicular damage, including Sertoli cell apoptosis, gonocyte abnormalities, germ cell depletion, and Leydig cell hyperplasia, with more severe effects at higher doses (300 and 600 mg/kg bw/day). Reduced organ weights (testis, prostate, seminal vesicles, epididymides), seminiferous tubule shrinkage, Sertoli cell vacuolization, and germinal epithelium depletion, particularly in oral dosing groups.	[14]
Eight-week-old male C57BL/6J mice	DEHP	1500 mg/kg/day by gavage for 14 days	Increased seminiferous tubule lumen size, decreased germ cell area, disrupted cellular regulation and autophagy, with elevated but non-significant collagen deposition, and no significant change in testis weight or cleaved caspase-3 expression.	[15]
Five-week-old male CD-1 mice	MPs + DEHP (low and high), MPs + DEHP, DBP, DEP, DMP mixture (low and high)	100 mg/kg/day by gavage for 30 days; DEHP (5 μg/L or 50 μg/L), PAE mixture (5 μg/L or 50 μg/L)	All treatments reduced sperm count, vitality, and testicular weight, increased oxidative stress, and disrupted spermatogenesis, with stronger effects in MPs + H-DEHP and MPs + H-MIX groups. Additionally, altered genes and pathways related to spermatogenesis and testicular health was obsereved, with some DEHP detected in the testis.	[16]
Four-week-old male Swiss Webster albino mice	DEP	3 mg/g/day by gavage for 54 days	Reduced testes weight, GSI, and sperm count, increased abnormal sperm morphology, and decreased serum testosterone while elevating LH levels. Also, severe testicular	[17]

			damage, including germ cell degeneration, seminiferous tubule disruption, Leydig and Sertoli cell damage, and reduced antioxidant capacity.	
Twenty-one-day-old female ICR mice	DEHP	2 g/kg/day by gavage for 8 days	Reduced ovarian weight, decreased primordial follicles, and increased atretic follicles, indicating impaired ovarian function. It lowered AMH levels (reduced ovarian reserve), suppressed granulosa cell (GC) proliferation (↓Ki67); and upregulated SLC39A5, activating the NF-κB/NLRP3 pathway, which triggered pyroptosis (↑cleaved GSDMD, Caspase-1, IL-1β) in GCs.	[18]
Three-week-old male ICR mice	DEHP	50, 200, 500 mg/kg/day by gavage for 4 weeks	Testicular atrophy, disrupted seminiferous tubule structure, and induced mitochondrial damage, BTB dysfunction, Sertoli cell dysfunction, and sperm abnormalities. It also increased oxidative stress and iron overload, activating ferroptosis.	[19]
Twenty-one-day-old male CD-1 mice	DEHP	2.4–3 μg/kg/day orally from postnatal day 21 to 45	Shortened anogenital distance and reduced seminal vesicle weight were observed, along with decreased sperm concentration, morphology, and acrosome integrity. No significant changes occurred in testes, epididymides, prostate weights, sperm viability, apoptosis, or testicular histology. Gene expression analysis showed downregulation of androgen synthesis, germ cell, Sertoli cell, oxidative stress-related, and other endocrine and apoptosis-related genes. Protein levels of 3β-HSD1/2 remained unchanged.	[20]
Ten- to twelve-week-old male Wistar rats	DEHP	1000 mg/kg/day by gavage for 2, 4, 6, and 8 weeks	Reduced testis weight and serum testosterone levels, disrupted seminiferous tubules, degenerated epithelium, reduced spermatogonia and Sertoli cells, and abnormal sperm morphology, worsening with longer exposure durations.	[21]
Twenty-one-day-old male Sprague-Dawley rats	DEHP	250, 500 mg/kg/day by gavage from postnatal day 22 to 35	Testicular damage, regressed seminiferous tubules, reduced testosterone levels, impaired spermatogenesis, enhanced apoptosis and altered RNA	[22]

			methylation, and increased oxidative stress.	
Three- to four-month-old male Wistar Albino rats	DBP	500 mg/kg/day by Intraperitoneal injection for 4 weeks	Testicular damage, sloughing of germ cells, vascular congestion, and reduced seminiferous tubule diameter and germinal epithelial thickness. Also, increased oxidative stress, reduced sperm motility and concentration, and increased abnormal sperm rates.	[23]
Five-day-old male C57BL/6 mice	DEHP	40, 80 µg/kg by Intraperitoneal injection for 10 days	Testicular damage, disrupted seminiferous tubule structure, impaired Sertoli cell maturation, and altered Leydig cell steroidogenesis. It induced apoptosis (40 µg/kg) or autophagy (80 µg/kg) in undifferentiated spermatogonia, BTB disruption, and altered mitochondrial function and acetyl-CoA levels, leading to histone hyperacetylation (40 µg/kg) or suppressed acetylation (80 µg/kg).	[24]
Twenty-one-day-old male Sprague-Dawley rats	DEHP	0, 500, or 1000 mg/kg/day by gavage for 56 days	Reduced testicular organ coefficient, caused atrophic seminiferous tubules, mitochondrial edema, ER swelling, and decreased sperm count. It also induced oxidative stress, apoptosis and ER stress.	[25]
Sixty-day-old female CD-1 mice	DBP	1, 10, 1000 mg/kg orally single dose or 10 consecutive days	MBP, a toxic DBP metabolite, was detected in the ovary in levels exceeding those in human follicular fluid. Ovarian biotransformation enzymes were expressed and altered in a time- and dose-specific manner, with Cyp1b1 upregulation, which is an enzyme involved in estrogen metabolism.	[26]
Twenty-one-day-old male C57BL/6 mice	DEHP	0, 250, 500 mg/kg/day by gavage from postnatal day 22 to 35	Seminiferous tubule regression, reduced epithelium thickness, and decreased serum testosterone levels. It also promoted apoptosis and inhibited Leydig cell proliferation, leading to testicular dysfunction.	[27]
Four-week-old male C57BL/6 mice	DEHP	1, 250, 500 mg/kg/day by gavage for 4 weeks	Testicular injury, including hypo-spermatogenesis and germ cell degeneration, with increased seminiferous tubule damage scores. It also induced telomere dysfunction, cellular senescence, and altered m6A modification,	[28]

			indicating epigenetic regulation of testicular aging and dysfunction.	
Newborn female Sprague-Dawley rats	DEHP	5, 500 mg/kg/day by gavage during postnatal days 22–28 or 22–70	Low-dose advanced puberty and increased reproductive hormones, while high-dose delayed puberty, disrupted estrous cycles, reduced hormones, and caused ovarian abnormalities, with dose-dependent effects on Kiss1 and GnRH expression.	[29]
Six-week-old male Sprague-Dawley rats	DBP	800 mg/kg/day by gavage for 2 weeks	Reduced sperm count, viability, and testosterone levels, while increasing FSH, LH, and sperm malformation rates. It also decreased AGD, testicular weight, and antioxidant markers, increased oxidative stress, and downregulated Nrf2 pathway proteins, causing testicular injury and impaired spermatogenesis.	[30]
Three-week-old male ICR mice	DEHP	1000 mg/kg/day orally for 6 weeks	Testicular atrophy, reduced seminal vesicle weight, shortened AGD, and reduced sperm count and motility. It also distorted seminiferous tubules, reduced spermatogenic cells, increased apoptosis, and decreased serum testosterone, estradiol, FSH, LH and zinc levels, and altered testicular mineral levels.	[31]
Six- to seven-week-old female Sprague-Dawley rats	DEHP	0, 5, 50, 100, 250, 500, 1000, 2000, 3000 mg/kg/day by gavage for 2 weeks	Disrupted estrous cycles, reduced AMH levels, and decreased uterine/ovarian mass, with upregulated steroid biosynthesis genes.	[32]
Eight-week-old male C57BL/6J mice	DBP	200 mg/kg/day by gavage for 28 days	Reduced sperm motility, increased abnormal sperm morphology, elevated testicular LPO levels, altered apoptosis markers, decreased Prnd and PRND levels, downregulated Sertoli cell markers, and upregulated immature markers. No significant changes were observed in organ weights, testis histology, DSP, or serum testosterone levels.	[33]
Three-week-old male ICR mice	DEHP	500 mg/kg/day by gavage for 4 weeks	Reduced testosterone, sperm motility, and increased abnormalities, elevated oxidative stress, decreased antioxidants, caused mitochondrial damage, downregulated spermatogenesis	[34]

			proteins, and disrupted fatty acid metabolism in the testes.	
Five-week-old male BALB/c mice	DBP	2.15 mg/kg/day by tail vein injection for 37 days	Increased ROS levels and altered expression of 83 proteins (47 upregulated, 36 downregulated), affecting translation initiation and RNA splicing, with minor germ cell disorganization in the testes. No significant changes were observed in sperm count, malformation rate, MDA and GSH levels, or most testicular structures.	[35]
Six-week-old male C57BL/6N mice	DBP	250 mg/kg/day by gavage for 28 days	Increased ROS, activated JAK2/STAT3, reduced MMP, and triggered mitophagy and apoptosis. It also decreased mitochondrial proteins, testosterone synthesis enzymes, and serum testosterone.	[36]
Two- to three-month-old female Wistar rats	DEHP	0, 300, 1000, 3000 mg/kg/day by gavage for 4 weeks	Prolonged estrous cycles, ovarian damage (follicular atresia, GC apoptosis), reduced hormone levels (LH, P4, FSH, T, E2), inhibited steroid receptors (PR, ER), downregulated steroidogenic enzymes, and increased the Bax/Bcl2 ratio were observed. No significant changes were found in caspase-3 activity, and organ coefficients were only reduced in the right ovary.	[37]
Thirty-five-day-old male Sprague-Dawley rats	DEHP	100, 500, 900 mg/kg by gavage for 7, 14, 21, 28, and 35 days	Dose- and time-dependently caused testicular damage, reduced testosterone, decreased steroidogenic enzymes, and lowered testicular weight and seminiferous epithelium layers.	[38]
Six-week-old male Sprague-Dawley rats	DEHP	0, 250, 500, 1000 mg/kg/day by gavage for 28 days	Reduced testis weight, sperm count, and testosterone with testicular damage, increased apoptosis, decreased antioxidant enzymes, and mitochondrial damage. MDA showed a non-significant increasing trend. Autophagy and apoptosis-related proteins were altered dose-dependently.	[39]
Ten-week-old male SPF ICR mice	DEHP	1 g/kg/d by gavage for 60 days	Reduced testis weight, sperm count, and serum testosterone, caused testicular damage (disrupted spermatogenic epithelium, vacuolization, abnormal spermatocytes), increased apoptosis, oxidative stress, and mitochondrial damage.	[40]

Postnatal day (PND) 21 male Sprague-Dawley rats	DEHP	0, 250, 500 mg/kg/day by gavage for 10 days	Dose-dependent testicular injury, disorganized seminiferous epithelium, reduced germ cells, and Sertoli cell deformation, increased oxidative stress, altered ER stress markers, activated mitophagy, and enhanced pyroptosis.	[41]
Thirty-nine-to forty-day-old female CD-1 mice	DEHP, DiNP	DEHP (20 µg/kg/day, 200 µg/kg/day, 20 mg/kg/day, 200 mg/kg/day), DiNP (20 µg/kg/day, 100 µg/kg/day, 20 mg/kg/day, 200 mg/kg/day) orally for 10 days	Both exposures disrupted estrous cyclicity, decreased primordial and primary follicles, reduced fertility (lower gestational index, litter size, and female pup percentage), and decreased estradiol, while only DiNP increased FSH and inhibin B but decreased testosterone.	[42]
Eight-week-old male C57BL/6J mice	DBP	200 mg/kg/day orally for 28 days	Reduced sperm motility and increased morphological defects without affecting daily sperm production or testis weight. Testicular oxidative stress and apoptosis markers were elevated. PRND expression was downregulated, contributing to sperm structural abnormalities. Sertoli cell dysfunction was evident, with decreased functional markers and increased immaturity markers. Serum testosterone levels remained unchanged.	[43]
Five-week-old male Sprague-Dawley rats	DEHP	500 mg/kg bw/day for 10 days by oral gavage	DEHP reduced Ventral prostate (VP) (-16.8%), Seminal vesicles (SV) (-13.6%), and Levator ani-bulbocavernosus muscle (LABC) (-15.3%) weights, but these decreases were not statistically significant.	[44]

Table S4: *Summary of epidemiological studies on phthalate exposure and reproductive health outcomes, including study design, population, phthalates detected, and key findings.*

**Study Design & Population**	**Phthalates Detected**	**Outcomes Measured**	**Main Findings**	**Reference**
C-C, 155 infertile men (30–67 years) & 211 fertile men (22–58 years) in Milan, Italy	MnBP, MEP (DEP metabolite), MBzP, MnOP, MEHP & MEHHP (DEHP metabolites)	Associations between urinary mPAEs with semen parameters & occupational and lifestyle factors	Infertile men had higher urinary phthalate levels (except MEP), with frequent perfume usage linked to higher DEP exposure. Among them, 90.07% had low sperm motility, 53.69% low total sperm count, 46.31% low sperm concentration, and 16.56% low semen volume.	[45]

			Occupational exposure in farming, dentistry, and artisanal work increased infertility risk.	
C-S, 76 women under 35 years undergoing IVF in Wuhan, China	MMP, MEP, MBP, MBzP, MEHP, MEHHP, MEOHP, MOP	Associations of mPAEs with inflammatory cytokines in the follicular fluid (FF)	MEP was positively associated with IL-6, and MEHHP showed a suggestive link with IL-6. MBzP, MEHP, and %MEHP were inversely associated with MCP-1, while phthalate mixtures were positively linked to TNF-α at lower percentiles.	[46]
C-S, 525 women (20-45 years) undergoing IVF in Wuhan, China	MMP, MEP, MBP, MBzP, MEHP, MEHHP, MEOHP, MOP	Associations between urinary mPAEs and antral follicle count (AFC)	Positive associations found between MBP, MEOHP, and ΣPAEs and AFC, with dose-response relationships. Higher tertiles showed 7.02%, 8.84%, and 6.19-9.09% increases, respectively. Older women (≥35 yrs) had stronger positive associations, while younger women (<35 yrs) showed inverse associations.	[47]
C-S, 451 males under 35 years in Wuhan, China	MMP, MEP, MBP, MBzP, MEHP, MEHHP, MEOHP, MOP	Associations between urinary mPAEs with urinary and serum hormones (T & E2) levels	Phthalate mixtures were inversely associated with serum T and E2 (MEHP major contributor) but positively associated with urinary T and E2 (MBzP major contributor). Low molecular weight phthalates had minimal effects, and no significant correlation was found between urinary and serum hormone levels.	[48]
C, 304 boys enrolled at ages 8–9 years in Chapaevsk, Russia	15 mPAEs, including anti-androgenic phthalates (AAPs) (DEHP, DINP, MBzP, MnBP, MiBP)	Association between prepubertal urinary concentrations of mPAEs and pubertal onset	Higher prepubertal exposure to AAPs was linked to delayed puberty in boys, with 8–14 months delay in pubarche and 5.4–8.3 months delay in genitalia/testicular development. Strongest effects occurred at intermediate exposure levels. Higher quartiles of non-AAPs (MEP, MCPP) showed delayed pubarche, but less consistent associations.	[49]
C-S, 37 women with diminished ovarian reserve (DOR) (32.7 ± 3.8 years) & 227 women with normal ovarian reserve (NOR) (32.0 ± 3.8 years) in Tianjin, China	MEP, MEOHP, MEHP, MBP, MIBP, MBZP, MCMHP, MECPP, MEHHP, MMP, MCPP	Association between mPAEs and diminished ovarian reserve (DOR) through the dysregulation of the steroids in follicular fluid (FF)	Androstenedione (A4), corticosterone (CORT), cortisol (COR), and cortisone were downregulated in women with diminished ovarian reserve (DOR). MEP, MEOHP, and MEHP were negatively associated with CORT and COR, while MEOHP, MEHP, MBP, and MiBP were positively associated with cortisone. MEP and MEOHP significantly contributed to the decline of COR and CORT, indirectly reducing retrieved oocyte number (RON). However, no significant difference in mPAE levels was found between DOR and NOR groups.	[50]

C, 72 women (30.9 ± 3.5 years) undergoing IVF	MBzP, MCPP, mECPP, MEHP, MEHHP, MEOHP, MEP, MiBP, MiNP, MnBP, MCOMHP, MCOMOP	Associations between mPAEs and reproductive hormone levels in follicular fluid (FF)	MiBP and MnBP were significantly associated with higher E2 levels, and no significant associations were found with progesterone, AMH, or inhibin B.	[51]
C-S, 975 women (20–44 years) [143 women (16.2%) reported infertility]	DEHP metabolites (MEHP, MEHHP, MEOHP, MECPP)	Association between urinary DEHP metabolites and infertility	Higher DEHP exposure (second and third quartiles) was linked to increased infertility odds, but the highest quartile showed no significant association.	[52]
C-S, 403 men (18–54 years) seeking fertility counseling in Shenzhen, China	MnBP (most prevalent at 96%)	Associations between mPAEs in seminal plasma with androgen synthesis and semen quality	MnBP was inversely associated with sperm concentration, count, and motility parameters in a dose-dependent manner, and positively associated with androstenedione (ADD) and pregnenolone (PGL) levels. ADD mediated 6.4–11.9% of the association between MnBP and reduced sperm motility.	[53]
C, 351 boys (mean age: 9.05 years) and 389 girls (mean age: 7.98 years) in Xiamen, China	MMP, MEP, MnBP, MiBP, MEHP, MEHHP, MEOHP	Associations between urinary mPAEs and sex hormones with early pubertal onset early pubertal onset	Persistent exposure to PAEs was positively associated with early pubertal onset in girls, while boys had higher phthalate exposure levels than girls. Persistent exposure to PAEs and E2 had synergistic associations with early pubertal onset in both sexes. Testosterone (TT) had antagonistic effects in boys, reducing early puberty onset.	[54]
C-S, 88 infertile men (29.2 ± 5.2 years) in Tianjin, China	11 mPAEs in urine and serum	Associations between PAE exposure and semen quality and hormone level outcomes	Serum levels of ∑mPAE had positive associations with sperm concentration, motility rate, and progressive motility, and negative associations with testosterone and LH levels. MCMHP was negatively correlated with FSH. FSH mediated MCMHP's effect on progressive motility, while LH mediated ∑mPAE's impact on sperm concentration and motility, indicating phthalates may disrupt fertility via hormonal and sperm changes.	[55]
C-S, 909 healthy men (22–45 years) in Hubei, China	9 mPAEs including MEHP, MEP, MBP, MEHHP	Associations between urinary mPAEs levels and sperm quality	mPAEs, especially MEP and MEHP, were negatively associated with sperm concentration and total sperm count. MEHP was also linked to reduced total and progressive motility. Sperm mitochondrial DNA copy number (mtDNAcn) mediated part of	[56]

			MEHP's effects. No significant mediation was found for sperm telomere length (TL), though higher TL was associated with lower sperm concentration and count.	
C-S, 441 women (30.6 ±4.2 years) seeking fertility assistance in Wuhan, China	8 mPAEs in urine and FF including MBP, MiBP, MECPP, MEHP	Associations between PAE exposure and menstrual cycle characteristics	Urinary MEHP was associated with a decrease of 0.20 days in menstrual bleeding duration, and FF MECPP & ΣDEHP were associated with a decrease in menstrual cycle length. No significant associations were found with irregular cycles, dysmenorrhea, or hypomenorrhea.	[33]
C-S, 20 adolescents (10-19 years) and 24 oocyte donors (22-30 years) in IL, USA	9 mPAEs including MBP, MiBP	Association between mPAEs in follicular fluid (FF) of adolescents and oocyte donors with ovarian reserve, cumulus cell transcriptome, and IVF outcomes	Adolescents had higher mPAEs levels, especially MBP and MiBP, compared to oocyte donors. In donors, higher FF levels were positively associated with antral follicle count (AFC). Adolescents with high mPAEs levels had 248 differentially expressed genes (DEGs) in cumulus cells involved in cell motility and development significantly downregulated and metabolic and catabolic processes upregulated. No significant associations were found between FF phthalate levels and IVF outcomes.	[57]
C-C, 191 infertile women and 95 fertile women (18–40 years) in Jordan	DEHP metabolites (MEHHP, MEOHP)	Association between urinary DEHP metabolites levels and fertility outcomes	Cases had higher urinary concentrations of MEOHP and total DEHP compared to controls. MEOHP was significantly linked to infertility. Key exposure sources included heating plastic in microwaves and using skin & eye make-up, sunscreen and nail polish. DEHP metabolite levels in Jordan were higher than in many other countries, except for MEHHP.	[58]
C-S, 297 women (20–39 years) in Tianjin, China	11 mPAEs including MiBP, MEHP, MEHHP, MEOHP, MCMHP	Association between serum mPAEs and biomarkers of ovarian reserve	MiBP was associated with a decline in antral follicle count (AFC), while MEHHP increased it. MEHP and MEOHP were negatively associated with estradiol (E2), while MCMHP increased it. MiBP was negatively associated with Anti-Müllerian Hormone (AMH) levels. Nonlinear associations: MEP increased FSH in the third quantile, while MCMHP decreased it also the third quantile. MEHHP increased AMH at high concentrations. Principal Component Analysis (PCA) showed that a component with high mBP, miBP, and	[59]

			mEHP levels was negatively associated with E2.	
C-C, 82 women with polycystic ovary syndrome PCOS and 359 controls (19–48 years) in Saudi Arabia	MEP, MnBP, MiBP, MBzP, MEHP, MEHHP, MEOHP, MECPP	Association between urinary mPAEs levels and polycystic ovary syndrome (PCOS)	Women with PCOS had significantly higher levels of MECPP, MEOHP, ∑DEHP, and ∑HMW (sum of high molecular weight phthalates MBzP, MEHP, MEHHP, MEOHP, MECPP). The odds of PCOS decreased by 44% with an increase in %MEHP (the ratio of MEHP to ∑DEHP). Urinary mPAEs levels were several-fold higher than in national surveys from other countries.	[60]
C-S, 138 women undergoing 179 fresh IVF cycles (32-37 years) in MA, USA	MEP, MBP, MEHP, MEHHP, MEOHP, MECPP	Association between urinary mPAEs levels and follicular fluid (FF) anti-Müllerian hormone (AMH) concentrations	Urinary MEOHP, MECPP, ∑DEHP were negatively associated with FF AMH concentrations, suggesting reduced ovarian reserve. No significant association was found between MEHP and FF AMH levels.	[61]
C, Initial: 516 boys (8-9 years) and final: 223 men (18–19 years) in Chapaevsk, Russia	(DEHP, DiNP and DiDP) metabolites + MnBP, MiBP, MBzP, MCPP	Associations between urinary mPAEs levels measured at four different time points (prepuberty, early puberty, late puberty, and sexual maturity) and semen quality	Higher urinary concentrations of ∑DiNP metabolites during late puberty were significantly associated with poorer semen quality, including 30% lower sperm concentration, 32% lower total sperm count, and 30% lower total progressive motile count compared to men with lower exposure. Higher urinary MiBP metabolite concentrations during early puberty showed a trend toward poorer semen quality.	[62]
C-C, 40 endometrial polyp patients and 80 controls (mean age: 31.3 years) in Wuhan, China	MEP, MBP, MiBP, MBzP, MEHHP, MEOHP, MECPP, MEHP	Associations between urinary mPAEs levels and the risk of endometrial polyp	Individual mPAEs (MBzP, MEHHP, MECPP, MEOHP, MEHP, and ∑DEHP) was associated with an increased risk of endometrial polyp, with ∑DEHP showing the strongest association. A one-quartile increase in phthalate mixtures was associated with a 3.14-fold increased risk of endometrial polyp. MEHHP, MBzP, MECPP, and MEHP were the main positive contributors.	[63]
C-S, 106 fertile men (18–39 years) & 44 infertile men (26–40 years) in Montreal, Canada	MEP, DEHP metabolites (MEHP, MEHHP, MEOHP, MECPP, MCMHP), MnBP, MiBP, MBzP, MCPP	Association between urinary mPAEs concentrations (free and conjugated-forms) and male fertility	Higher MEP and DEHP metabolite concentrations were found in infertile men, but only conjugated-form MEP and MEHP showed significant differences. MBzP levels were lower in infertile men, contrary to previous studies. Strong correlations were observed between DEHP and mid-size phthalate metabolites, while MEP and MCPP showed little correlation.	[64]

			Infertile men had higher phase II biotransformation efficiency, indicated by lower free-to-conjugated ratios for MEP and MEHP. Most individual DEHP metabolites showed no significant differences between groups.	
C-C, reproductive-aged 562 women (96 PCO cases, 96 PCOS cases, and 370 controls) in Wuhan, China	MEP, MiBP, MBP, MBzP & DEHP metabolites (MEHHP, MEOHP, MECPP, MEHP)	Associations between urinary mPAEs and polycystic ovary (PCO) and polycystic ovary syndrome (PCOS)	MiBP, MBzP, and the molar sum of DEHP metabolites (∑DEHP) were associated with increased prevalence of PCO, and MBzP, MEHP, and ∑DEHP were associated with increased prevalence of PCOS. Mixtures of phthalate metabolites were positively associated with both PCO and PCOS, with MBzP was the most significant contributor to both PCO and PCOS.	[65]
C-S, 22 infertile men and 78 fertile men (20–45 years) in Padova, Italy	DNOP (most signficant), DEHP, DEP, DBP	Association between PAEs levels in semen and sperm function	DNOP was significantly more prevalent in the semen of infertile men, and was linked to lower acrosome reaction (AR) levels. PAEs levels were higher in men with idiopathic infertility, suggesting a link between exposure and impaired sperm function.	[66]
C-C, 107 women with endometriosis (33.6 ± 6.7 years) and 70 healthy women (32.6 ± 6.8 years) in Fujian, China	DMP, MMP, DEP, MEP, DBP, MBP, BBzP, MBzP, DEHP, MEHP	Association between phthalate exposure and endometriosis	Endometriosis patients had higher urinary levels of phthalates, especially DEHP, which significantly increased endometriosis risk. The combination of DEHP, MEHP, and MBP showed high diagnostic potential for endometriosis. Endometriosis patients also had higher rates of infertility and dysmenorrhea, lower pregnancy success, and reported higher use of plastic products and cosmetics.	[32]
C-S, 111 men (31.4 ± 4.7 years) in Wuhan, China	8 mPAEs including MEHP, MEHHP, MBP	Associations between urinary mPAEs levels with sperm protamine levels and spermatozoa apoptosis	MEHP was positively associated with protamine-1 and protamine ratio, with higher exposure linked to increased levels. Protamine-2 was inversely related to viable sperm cells (Annexin V+/PI-), while protamine ratio was positively associated with apoptotic sperm cells (Annexin V+/PI+). Protamine ratio mediated 55.6% of the association between MEHP and increased sperm apoptosis.	[67]
C-S, 111 men in Wuhan, China	Urine & seminal plasma: MMP, MEP, MBP, MEHP, MEHHP,	Associations between phthalate exposures and spermatogenesis-related	Urinary and seminal plasma MEHP exposure was associated with significant decreases in spermatogenesis-related miRNA106a. Mixtures of seminal plasma phthalates above the 35th	[68]

	MEOHP. Urine only (MBzP, MOP)	miRNA106a levels in seminal plasma.	percentile also reduced miRNA106a, with MEHP being the primary contributor.	
C-C, 173 women with premature ovarian failure (POF) 246 control (33.2 ± 5.9 years) in Zhejiang, China	MMP, MEP, MiBP, MnBP, MBzP, MEHP, MEOHP, MEHHP	Associations between urinary mPAEs levels and premature ovarian failure (POF)	Higher urinary levels of MiBP were linked to a 38% greater likelihood of premature ovarian failure (POF). Phthalate exposure was linked to lower estradiol, higher FSH, and a reduced estradiol/FSH ratio. MiBP had the highest median concentration, while MBzP had the lowest, and the sum of all phthalates was similar in controls and cases.	[69]
C, 333 male partners of couples trying to conceive (31.9 ± 4.8 years) in Michigan and Texas, USA	MEHP, MEOHP, MEHHP, MECPP, MCMHP, MBzP, MCPP, MMP, MEP, MBP, MiBP	Association between urinary mPAEs and sperm epigenetic aging (SEA)	Nine out of eleven phthalate metabolites (82%) showed positive trends with advanced SEA, specifically MEHHP, MiBP, and MMP. Phthalate mixtures, driven by MiBP, MMP, and MBzP, were positively associated with SEA.	[70]
C, 105 women undergoing IVF (30.7 ± 3.7 years)	11 mPAEs including MCOMHP, MECPP, MnBP, MBzP, MiBP, MEP, MCOMOP	Association between mPAEs levels in follicular fluid (FF) and the expression of extracellular vesicle microRNAs (EV-miRNAs)	mPAEs in FF were associated with altered expression of extracellular vesicle microRNAs (EV-miRNAs), which regulate pathways critical for oocyte development, maturation, and fertilization. Higher levels of MEP were linked to poorer embryo development, and lower levels of MEOHP were associated with successful fertilization.	[71]
C, 641 women who underwent IVF/ICSI treatment (30.6 ± 4.1 years) in Wuhan, China	MEP, MiBP, MBP, MBzP, MEHP, MEHHP, MEOHP, MECPP	Associations between mPAEs levels in follicular fluid (FF) and reproductive outcomes	MBzP, MEHHP, and MEHP were inversely associated with oocyte yield, mature oocyte number, and 2PN zygote number, while $\sum \mathrm{DEHP}$ reduced oocyte and mature oocyte numbers. MEP showed a positive association with oocyte outcomes. Phthalate mixtures were also linked to reduced oocyte yield. No significant associations were found with fertilization rate.	[72]

Legend: C: Cohort, C-S: Cross-Sectional, C-C: Case-Control, PAEs: Phthalic acid esters, mPAEs: Monophthalic acid esters (phthalate metabolites).

Abbreviations

AGD: Anogenital Distance

AFC: Antral Follicle Count

AMH: Anti-Müllerian Hormone

BTB: Blood-Testis Barrier

CAT: Catalase

Caspase-1: Cysteine-aspartic acid protease 1

ER: Endoplasmic Reticulum

FF: Follicular Fluid

FSH: Follicle-Stimulating Hormone

GC: Granulosa Cells

GSDMD: Gasdermin D

GSH: Glutathione

GSI: Gonadosomatic Index

IL-1β: Interleukin-1 beta

IL-6: Interleukin-6

IVF: *In vitro* Fertilization

JAK2/STAT3: Janus kinase 2/Signal transducer and activator of transcription 3

LH: Luteinizing Hormone

MDA: Malondialdehyde

m6A: N6-methyladenosine

mtDNAcn: Mitochondrial DNA Copy Number

NF-κB: Nuclear Factor kappa-light-chain-enhancer of activated B cells

NLRP3: NLR Family Pyrin Domain Containing 3

Nrf2: Nuclear factor erythroid 2-related factor 2

PCOS: Polycystic Ovary Syndrome

ROA: Route of Administration

ROS: Reactive Oxygen Species

SLC39A5: Solute Carrier Family 39 Member 5

TNF-α: Tumor Necrosis Factor-alpha

PRND: Prion-like Protein Doppel

PLZF: Promyelocytic Leukemia Zinc Finger

References

[1] B. Xu *et al.*, “Activation of the p62-Keap1-Nrf2 pathway protects against oxidative stress and excessive autophagy in ovarian granulosa cells to attenuate DEHP-induced ovarian impairment in mice,” *Ecotoxicol Environ Saf*, vol. 265, p. 115534, Oct. 2023, doi: 10.1016/j.ecoenv.2023.115534.

[2] L.-Z. Xia *et al.*, “Alleviative effect of quercetin against reproductive toxicity induced by chronic exposure to the mixture of phthalates in male rats,” *Ecotoxicol Environ Saf*, vol. 270, p. 115920, Jan. 2024, doi: 10.1016/j.ecoenv.2023.115920.

[3] Y. Zhao *et al.*, “Connexin-43 is a promising target for lycopene preventing phthalate-induced spermatogenic disorders,” *J Adv Res*, vol. 49, pp. 115–126, Jul. 2023, doi: 10.1016/j.jare.2022.09.001.

[4] Sajida Batool *et al.*, “Curative Potentials of Garlic (Allium sativum) Extract against Di-(2-Ethylhexyl) Phthalate Induced Reproductive Toxicity in Female Mice,” *Proceedings of the Pakistan Academy of Sciences: B. Life and Environmental Sciences*, vol. 59, no. 3, pp. 39–53, Sep. 2022, doi: 10.53560/PPASB(59-3)720.

[5] A. Anis *et al.*, “Cytoprotective potency of naringin against di-n-butylphthalate (DBP)-induced oxidative testicular damage in male rats,” *Naunyn Schmiedebergs Arch Pharmacol*, vol. 397, no. 6, pp. 4309–4319, Jun. 2024, doi: 10.1007/s00210-023-02874-y.

[6] J.-C. Liu *et al.*, “DEHP exposure to lactating mice affects ovarian hormone production and antral follicle development of offspring,” *J Hazard Mater*, vol. 416, p. 125862, Aug. 2021, doi: 10.1016/j.jhazmat.2021.125862.

[7] L. Yang *et al.*, “DEHP induces ferroptosis in testes via p38α-lipid ROS circulation and destroys the BTB integrity,” *Food and Chemical Toxicology*, vol. 164, p. 113046, Jun. 2022, doi: 10.1016/j.fct.2022.113046.

[8] B.-B. Zhu *et al.*, “Di-(2-ethylhexyl) phthalate induces testicular endoplasmic reticulum stress and germ cell apoptosis in adolescent mice,” *Environmental Science and Pollution Research*, vol. 28, no. 17, pp. 21696–21705, May 2021, doi: 10.1007/s11356-020-12210-z.

[9] L. XueXia *et al.*, “Di-2-ethylhexyl phthalate (DEHP) exposure induces sperm quality and functional defects in mice,” *Chemosphere*, vol. 312, p. 137216, Jan. 2023, doi: 10.1016/j.chemosphere.2022.137216.

[10] A. M. Safar *et al.*, “Dietary exposure to an environmentally relevant phthalate mixture alters follicle dynamics, hormone levels, ovarian gene expression, and pituitary gene expression in female mice,” *Reproductive Toxicology*, vol. 122, p. 108489, Dec. 2023, doi: 10.1016/j.reprotox.2023.108489.

[11] E. Reyes-Cruz *et al.*, “Disruption of gonocyte development following neonatal exposure to di (2-ethylhexyl) phthalate,” *Reprod Biol*, vol. 24, no. 2, p. 100877, Jun. 2024, doi: 10.1016/j.repbio.2024.100877.

[12] V. Emojevwe *et al.*, “Duration-dependent effects of high dose of phthalate exposure on semen quality in adult male rats,” *JBRA Assist Reprod*, vol. 26, no. 1, 2022, doi: 10.5935/1518-0557.20210033.

[13] Y. Zhao *et al.*, “Effect of mitochondrial quality control on the lycopene antagonizing DEHP-induced mitophagy in spermatogenic cells,” *Food Funct*, vol. 11, no. 7, pp. 5815–5826, 2020, doi: 10.1039/D0FO00554A.

[14] L. Camacho, J. R. Latendresse, L. Muskhelishvili, C. D. Law, and K. B. Delclos, “Effects of intravenous and oral di(2-ethylhexyl) phthalate (DEHP) and 20% Intralipid vehicle on neonatal rat testis, lung, liver, and kidney,” *Food and Chemical Toxicology*, vol. 144, p. 111497, Oct. 2020, doi: 10.1016/j.fct.2020.111497.

[15] X. Zhou *et al.*, “Effects of Lycium barbarum glycopeptide on renal and testicular injury induced by di(2-ethylhexyl) phthalate,” *Cell Stress Chaperones*, vol. 27, no. 3, pp. 257–271, May 2022, doi: 10.1007/s12192-022-01266-0.

[16] Y. Deng, Z. Yan, R. Shen, Y. Huang, H. Ren, and Y. Zhang, "Enhanced reproductive toxicities induced by phthalates contaminated microplastics in male mice (Mus musculus)," *J Hazard Mater*, vol. 406, p. 124644, Mar. 2021, doi: 10.1016/j.jhazmat.2020.124644.

[17] C. Ara *et al.*, "Evaluation of sex steroid hormones and reproductive irregularities in diethyl phthalate-exposed premature mice: modulatory effect of raw honey against potential anomalies," *Environmental Science and Pollution Research*, vol. 28, no. 39, pp. 55265–55276, Oct. 2021, doi: 10.1007/s11356-021-14774-w.

[18] J. Sun, L. Gan, S. Lv, T. Wang, C. Dai, and J. Sun, "Exposure to Di-(2-Ethylhexyl) phthalate drives ovarian dysfunction by inducing granulosa cell pyroptosis via the SLC39A5/NF-κB/NLRP3 axis," *Ecotoxicol Environ Saf*, vol. 252, p. 114625, Mar. 2023, doi: 10.1016/j.ecoenv.2023.114625.

[19] Y. Zhao *et al.*, "Ferroptosis is critical for phthalates driving the blood-testis barrier dysfunction via targeting transferrin receptor," *Redox Biol*, vol. 59, p. 102584, Feb. 2023, doi: 10.1016/j.redox.2022.102584.

[20] P. Dostalova *et al.*, "Gestational and pubertal exposure to low dose of di-(2-ethylhexyl) phthalate impairs sperm quality in adult mice," *Reproductive Toxicology*, vol. 96, pp. 175–184, Sep. 2020, doi: 10.1016/j.reprotox.2020.06.014.

[21] K. Gouri and P. Sinha, "Impact of DEHP Toxicity on Testis of Rat Leading to Disruption of Testis Histology," *Toxicol Int*, pp. 507–514, Feb. 2023, doi: 10.18311/ti/2022/v29i4/30294.

[22] T.-X. Zhao *et al.*, "Increased m6A RNA modification is related to the inhibition of the Nrf2-mediated antioxidant response in di-(2-ethylhexyl) phthalate-induced prepubertal testicular injury," *Environmental Pollution*, vol. 259, p. 113911, Apr. 2020, doi: 10.1016/j.envpol.2020.113911.

[23] N. Başak Türkmen, İ. Ayhan, A. Taşlıdere, M. Aydın, and O. Çiftçi, "Investigation of protective effect of ellagic acid in phthalates-induced reproductive damage," *Drug Chem Toxicol*, vol. 45, no. 4, pp. 1652–1659, Jul. 2022, doi: 10.1080/01480545.2020.1853764.

[24] Y. Tian *et al.*, "Mechanisms of imbalanced testicular homeostasis in infancy due to aberrant histone acetylation in undifferentiated spermatogonia under different concentrations of Di(2-ethylhexyl) phthalate (DEHP) exposure," *Environmental Pollution*, vol. 347, p. 123742, Apr. 2024, doi: 10.1016/j.envpol.2024.123742.

[25] H. Zhang *et al.*, “Mitochondrial dysfunction and endoplasmic reticulum stress induced by activation of PPARα leaded testicular to apoptosis in SD rats explored to di-(2-ethylhexyl) phthalate (DEHP),” *Ecotoxicol Environ Saf*, vol. 268, p. 115711, Dec. 2023, doi: 10.1016/j.ecoenv.2023.115711.

[26] E. J. Jauregui, J. Lock, L. Rasmussen, and Z. R. Craig, “Mono- *n* -Butyl Phthalate Distributes to the Mouse Ovary and Liver and Alters the Expression of Phthalate-Metabolizing Enzymes in Both Tissues,” *Toxicological Sciences*, vol. 183, no. 1, pp. 117–127, Aug. 2021, doi: 10.1093/toxsci/kfab085.

[27] J. Wang *et al.*, “Multiple transcriptomic profiling: p53 signaling pathway is involved in DEHP-induced prepubertal testicular injury via promoting cell apoptosis and inhibiting cell proliferation of Leydig cells,” *J Hazard Mater*, vol. 406, p. 124316, Mar. 2021, doi: 10.1016/j.jhazmat.2020.124316.

[28] X. Zhu, H. Fu, J. Sun, Q. Di, and Q. Xu, “N6-methyladenosine modification on Hmbox1 is related to telomere dysfunction in DEHP-induced male reproductive injury,” *Life Sci*, vol. 309, p. 121005, Nov. 2022, doi: 10.1016/j.lfs.2022.121005.

[29] Z. Yu *et al.*, “Opposite effects of high- and low-dose di-(2-ethylhexyl) phthalate (DEHP) exposure on puberty onset, oestrous cycle regularity and hypothalamic kisspeptin expression in female rats,” *Reprod Fertil Dev*, vol. 32, no. 6, p. 610, 2020, doi: 10.1071/RD19024.

[30] M. Tang *et al.*, “Overexpression of miR-506-3p Aggravates DBP-Induced Testicular Oxidative Stress in Rats by Downregulating ANXA5 via Nrf2/HO-1 Signaling Pathway,” *Oxid Med Cell Longev*, vol. 2020, pp. 1–13, Nov. 2020, doi: 10.1155/2020/4640605.

[31] Z. Lu, Q. Huang, F. Chen, E. Li, H. Lin, and X. Qin, “Oyster Peptide-Zinc Complex Ameliorates Di-(2-ethylhexyl) Phthalate-Induced Testis Injury in Male Mice and Improving Gut Microbiota,” *Foods*, vol. 13, no. 1, p. 93, Dec. 2023, doi: 10.3390/foods13010093.

[32] H. Yi *et al.*, “Phthalate exposure and risk of ovarian dysfunction in endometriosis: human and animal data,” *Front Cell Dev Biol*, vol. 11, Jul. 2023, doi: 10.3389/fcell.2023.1154923.

[33] J. Li *et al.*, “Phthalate metabolites in urine and follicular fluid in relation to menstrual cycle characteristics in women seeking fertility assistance,” *Environ Int*, vol. 183, p. 108362, Jan. 2024, doi: 10.1016/j.envint.2023.108362.

[34] Y. Zhao *et al.*, “Phthalate-induced testosterone/androgen receptor pathway disorder on spermatogenesis and antagonism of lycopene,” *J Hazard Mater*, vol. 439, p. 129689, Oct. 2022, doi: 10.1016/j.jhazmat.2022.129689.

[35] X. Ye, T. Zhou, Y. Qin, S. He, H. Zhang, and S. Ding, “Reproductive toxicity of dibutyl phthalate adsorbed on carbon nanotubes in male Balb/C mice,” *Reproductive Toxicology*, vol. 110, pp. 180–187, Jun. 2022, doi: 10.1016/j.reprotox.2022.04.008.

[36] Q. Wang *et al.*, “Role of ROS/JAK2/STAT3 signaling pathway in di-n-butyl phthalate-induced testosterone synthesis inhibition and antagonism of lycopene,” *Food and Chemical Toxicology*, vol. 175, p. 113741, May 2023, doi: 10.1016/j.fct.2023.113741.

[37] N. Li, L. Zhou, J. Zhu, T. Liu, and L. Ye, “Role of the 17β-hydroxysteroid dehydrogenase signalling pathway in di-(2-ethylhexyl) phthalate-induced ovarian dysfunction: An in vivo study,” *Science of The Total Environment*, vol. 712, p. 134406, Apr. 2020, doi: 10.1016/j.scitotenv.2019.134406.

[38] L. Yang *et al.*, “The dynamic assessment of toxicity and pathological process of DEHP in germ cells of male Sprague Dawley rats,” *Reprod Biol*, vol. 20, no. 4, pp. 465–473, Dec. 2020, doi: 10.1016/j.repbio.2020.07.005.

[39] G. Fu *et al.*, “The role of STAT3/p53 and PI3K-Akt-mTOR signaling pathway on DEHP-induced reproductive toxicity in pubertal male rat,” *Toxicol Appl Pharmacol*, vol. 404, p. 115151, Oct. 2020, doi: 10.1016/j.taap.2020.115151.

[40] C. Zhang *et al.*, “Transcriptomic and proteomic characteristics of the di(2-ethylhexyl) phthalate-induced sperm dna damage mouse model,” *Hum Exp Toxicol*, vol. 41, Jan. 2022, doi: 10.1177/09603271221139444.

[41] Y. Hong *et al.*, “X-box binding protein 1 caused an imbalance in pyroptosis and mitophagy in immature rats with di-(2-ethylhexyl) phthalate-induced testis toxicity,” *Genes Dis*, vol. 11, no. 2, pp. 935–951, Mar. 2024, doi: 10.1016/j.gendis.2023.02.030.

[42] C. Chiang, L. R. Lewis, G. Borkowski, and J. A. Flaws, “Late-life consequences of short-term exposure to di(2-ethylhexyl) phthalate and diisononyl phthalate during adulthood in female mice,” *Reproductive Toxicology*, vol. 93, pp. 28–42, Apr. 2020, doi: 10.1016/j.reprotox.2019.12.006.

[43] J.-H. Lee, S. H. Park, C. Ryou, and M. C. Gye, "Phthalate plasticizer decreases the prion-like protein doppel essential for structural integrity and function of spermatozoa," *Ecotoxicol Environ Saf*, vol. 246, p. 114159, Nov. 2022, doi: 10.1016/j.ecoenv.2022.114159.

[44] C. R. Sung, H. G. Kang, J. Y. Hong, and S. J. Kwack, "Citrate ester substitutes for di-2-ethylhexyl phthalate: *In vivo* reproductive and *in vitro* cytotoxicity assessments," *J Toxicol Environ Health A*, vol. 83, no. 17–18, pp. 589–595, Sep. 2020, doi: 10.1080/15287394.2020.1798832.

[45] L. Caporossi *et al.*, "A Case–Control Study on the Effects of Plasticizers Exposure on Male Fertility," *Int J Environ Res Public Health*, vol. 20, no. 1, p. 235, Dec. 2022, doi: 10.3390/ijerph20010235.

[46] Y. Wang *et al.*, "Associations between phthalate metabolites and cytokines in the follicular fluid of women undergoing in vitro fertilization," *Ecotoxicol Environ Saf*, vol. 267, p. 115616, Nov. 2023, doi: 10.1016/j.ecoenv.2023.115616.

[47] Y. Yao, Y. Du, N. Guo, F. Liu, T. Deng, and Y. Li, "Associations between urinary phthalate concentrations and antral follicle count among women undergoing in vitro fertilization," *Front Endocrinol (Lausanne)*, vol. 14, Jan. 2024, doi: 10.3389/fendo.2023.1286391.

[48] M. Tian *et al.*, "Associations of environmental phthalate exposure with male steroid hormone synthesis and metabolism: An integrated epidemiology and toxicology study," *J Hazard Mater*, vol. 436, p. 129213, Aug. 2022, doi: 10.1016/j.jhazmat.2022.129213.

[49] J. S. Burns *et al.*, "Associations of prepubertal urinary phthalate metabolite concentrations with pubertal onset among a longitudinal cohort of boys," *Environ Res*, vol. 212, p. 113218, Sep. 2022, doi: 10.1016/j.envres.2022.113218.

[50] Y. Li *et al.*, "Dysregulation of steroid metabolome in follicular fluid links phthalate exposure to diminished ovarian reserve of childbearing-age women," *Environmental Pollution*, vol. 330, p. 121730, Aug. 2023, doi: 10.1016/j.envpol.2023.121730.

[51] N. Hoffmann-Dishon *et al.*, "Endocrine-disrupting chemical concentrations in follicular fluid and follicular reproductive hormone levels," *J Assist Reprod Genet*, vol. 41, no. 6, pp. 1637–1642, Jun. 2024, doi: 10.1007/s10815-024-03101-0.

[52] B. Trnka, M. Polan, and V. A. Zigmont, "Exposure to Di-2-ethylhexyl phthalate (DEHP) and infertility in women, NHANES 2013-2016," *Reproductive Toxicology*, vol. 103, pp. 46–50, Aug. 2021, doi: 10.1016/j.reprotox.2021.05.010.

[53] G. Yuan *et al.*, "Inverse association of certain seminal phthalate metabolites with semen quality may be mediated by androgen synthesis: A cross-sectional study from the South China," *Environ Int*, vol. 151, p. 106459, Jun. 2021, doi: 10.1016/j.envint.2021.106459.

[54] J. Liu *et al.*, "Persistent high exposure to exogenous phthalates and endogenous sex hormones associated with early pubertal onset among children: A 3.5-year longitudinal cohort study in China," *Ecotoxicol Environ Saf*, vol. 262, p. 115199, Sep. 2023, doi: 10.1016/j.ecoenv.2023.115199.

[55] B. Wang *et al.*, "Phthalate exposure and semen quality in infertile male population from Tianjin, China: Associations and potential mediation by reproductive hormones," *Science of The Total Environment*, vol. 744, p. 140673, Nov. 2020, doi: 10.1016/j.scitotenv.2020.140673.

[56] P. Yang *et al.*, "Phthalate exposure with sperm quality among healthy Chinese male adults: The role of sperm cellular function," *Environmental Pollution*, vol. 331, p. 121755, Aug. 2023, doi: 10.1016/j.envpol.2023.121755.

[57] D. Gokyer, M. J. Laws, A. Kleinhans, J. K. Riley, J. A. Flaws, and E. Babayev, "Phthalates are detected in the follicular fluid of adolescents and oocyte donors with associated changes in the cumulus cell transcriptome," Apr. 2024, doi: 10.1101/2024.04.04.588126.

[58] N. Abdo, H. Al-Khalaileh, M. Alajlouni, J. Hamadneh, and A. M. Alajlouni, "Screening for phthalates biomarkers and its potential role in infertility outcomes in Jordan," *J Expo Sci Environ Epidemiol*, vol. 33, no. 2, pp. 273–282, Mar. 2023, doi: 10.1038/s41370-022-00517-7.

[59] Y. Li *et al.*, "The association of serum phthalate metabolites with biomarkers of ovarian reserve in women of childbearing age," *Ecotoxicol Environ Saf*, vol. 242, p. 113909, Sep. 2022, doi: 10.1016/j.ecoenv.2022.113909.

[60] I. Al-Saleh, "The relationship between urinary phthalate metabolites and polycystic ovary syndrome in women undergoing in vitro fertilization: Nested case-control study," *Chemosphere*, vol. 286, p. 131495, Jan. 2022, doi: 10.1016/j.chemosphere.2021.131495.

[61] C. R. Sacha *et al.*, “Urinary phthalate metabolite concentrations are negatively associated with follicular fluid anti-müllerian hormone concentrations in women undergoing fertility treatment,” *Environ Int*, vol. 157, p. 106809, Dec. 2021, doi: 10.1016/j.envint.2021.106809.

[62] L. Mínguez-Alarcón *et al.*, “Urinary phthalate metabolite concentrations during four windows spanning puberty (prepuberty through sexual maturity) and association with semen quality among young Russian men,” *Int J Hyg Environ Health*, vol. 243, p. 113977, Jun. 2022, doi: 10.1016/j.ijheh.2022.113977.

[63] M. Zhang *et al.*, “Urinary phthalate metabolites and the risk of endometrial polyp: A pilot study from the TREE cohort,” *Environmental Pollution*, vol. 317, p. 120711, Jan. 2023, doi: 10.1016/j.envpol.2022.120711.

[64] Y.-L. Feng *et al.*, “Correlations of phthalate metabolites in urine samples from fertile and infertile men: Free-form concentration vs. conjugated-form concentration,” *Environmental Pollution*, vol. 263, p. 114602, Aug. 2020, doi: 10.1016/j.envpol.2020.114602.

[65] M. Zhang *et al.*, “Individual and joint associations of urinary phthalate metabolites with polycystic ovary and polycystic ovary syndrome: Results from the TREE cohort,” *Environ Toxicol Pharmacol*, vol. 102, p. 104233, Sep. 2023, doi: 10.1016/j.etap.2023.104233.

[66] I. Cosci *et al.*, “Lipophilic phthalic acid esters impair human sperm acrosomal reaction through the likely inhibition of phospholipase A2-signaling pathway,” *Biochem Pharmacol*, vol. 205, p. 115249, Nov. 2022, doi: 10.1016/j.bcp.2022.115249.

[67] F. Xiong *et al.*, “The Association of Certain Seminal Phthalate Metabolites on Spermatozoa Apoptosis: An Exploratory Mediation Analysis via Sperm Protamine,” *Environmental Pollution*, vol. 300, p. 118969, May 2022, doi: 10.1016/j.envpol.2022.118969.

[68] F.-P. Cui *et al.*, “Urinary and seminal plasma concentrations of phthalate metabolites in relation to spermatogenesis-related miRNA106a among men from an infertility clinic,” *Chemosphere*, vol. 288, p. 132464, Feb. 2022, doi: 10.1016/j.chemosphere.2021.132464.

[69] M. Cao, W. Pan, X. Shen, C. Li, J. Zhou, and J. Liu, “Urinary levels of phthalate metabolites in women associated with risk of premature ovarian failure and reproductive hormones,” *Chemosphere*, vol. 242, p. 125206, Mar. 2020, doi: 10.1016/j.chemosphere.2019.125206.

[70] O. A. Oluwayiose *et al.*, “Urinary phthalate metabolites and their mixtures are associated with advanced sperm epigenetic aging in a general population,” *Environ Res*, vol. 214, p. 114115, Nov. 2022, doi: 10.1016/j.envres.2022.114115.

[71] Z. Barnett-Itzhaki *et al.*, “Association between follicular fluid phthalate concentrations and extracellular vesicle microRNAs expression,” *Human Reproduction*, vol. 36, no. 6, pp. 1590–1599, May 2021, doi: 10.1093/humrep/deab063.

[72] W. Yao *et al.*, “Associations between Phthalate Metabolite Concentrations in Follicular Fluid and Reproductive Outcomes among Women Undergoing *in Vitro* Fertilization/Intracytoplasmic Sperm Injection Treatment,” *Environ Health Perspect*, vol. 131, no. 12, Dec. 2023, doi: 10.1289/EHP11998.
